# Expression of Ripk1 and DAM genes correlates with severity and progression of Krabbe disease

**DOI:** 10.1093/hmg/ddab159

**Published:** 2021-06-25

**Authors:** María B Cachón-González, Susan Wang, Timothy M Cox

**Affiliations:** Department of Medicine, University of Cambridge, Level 5, PO Box 157, Cambridge CB2 0QQ, UK; Department of Medicine, University of Cambridge, Level 5, PO Box 157, Cambridge CB2 0QQ, UK

## Abstract

Krabbe disease, an inherited leukodystrophy, is a sphingolipidosis caused by deficiency of β-galactocerebrosidase: it is characterized by myelin loss, and pathological activation of macrophage/microglia and astrocytes. To define driving pathogenic factors, we explored the expression repertoire of candidate neuroinflammatory genes: upregulation of receptor interacting protein kinase 1 (*Ripk1*) and disease-associated microglia (DAM) genes, including *Cst7* and *Ch25h*, correlated with severity of Krabbe disease genetically modelled in the twitcher mouse. Upregulation of *Ripk1* in Iba1/Mac2-positive microglia/macrophage associated with the pathognomic hypertrophic/globoid phenotype of this disease. Widespread accumulation of ubiquitinin1 in white and grey matter co-localised with p62. In Sandhoff disease, another sphingolipid disorder, neuroinflammation, accumulation of p62 and increased Ripk1 expression was observed. The upregulated DAM genes and macrophage/microglia expression of *Ripk1* in the authentic model of Krabbe disease strongly resemble those reported in Alzheimer disease associating with disturbed autophagosomal/lysosomal homeostasis. Activation of this shared molecular repertoire, suggests the potential for therapeutic interdiction at a common activation step, irrespective of proximal causation. To clarify the role of Ripk1 in the pathogenesis of Krabbe disease, we first explored the contribution of its kinase function, by intercrossing twitcher and the K45A kinase-dead Ripk1 mouse and breeding to homozygosity. Genetic ablation of Ripk1 kinase activity neither altered the neuropathological features nor the survival of twitcher mice. We conclude that Ripk1 kinase-dependent inflammatory and degenerative capabilities play no instrumental role in Krabbe disease; however, putative kinase-independent functions of Ripk1 remain formally to be explored in its molecular pathogenesis.

## Introduction

Krabbe disease or globoid cell leukodystrophy is a sphingolipidosis that inflicts a fulminant and painful disease course in young children; later onset forms also occur. Deficiency of the lysosomal enzyme β-galactosylceramidase [GALC; EC 3.2.1.46; (1)], and more rarely saposin A (2–4), a sphingolipid activator protein, cause this hereditary disorder in man and other mammals (5). GALC in concert with saposin A cleaves galactose from galactoceramide (GalCer), galactosylsphingosine [psychosine; (6)] and related galactolipids (7,8). Because Galcer is greatly enriched in myelin and myelin-producing cells (9), when the turnover of this lipid is impaired, myelin homeostasis is lost and disease ensues. Psychosine cytotoxicity was identified soon after its discovery (10–15), and recently its long-contended origin by either *de novo* synthesis or degradation has been resolved in the murine Krabbe disease model twitcher (16,17). Results implicate psychosine in the acute pathological changes and rapid death that typifies twitcher, but the molecular cascades downstream from psychosine that drive the acute neurodegeneration and inflammatory features of the disease are unknown. Emerging evidence indicates that GALC is also a mandatory requirement in other neural cell types (18,19).

Inflammation is a common feature of neurodegenerative diseases and macrophage/microglia play a key role in this process: after a demyelinating event, they facilitate remyelination by phagocytosing myelin debris after which it undergoes breakdown in lysosomes (19). The ability of microglia to mitigate some of the consequences of Krabbe disease has been shown in twitcher mice since experimental reduction of this cell population induces a more severe disease (20). A subtype of microglia termed disease associated microglia or degeneration-associated microglia (DAM) has been associated with several neurodegenerative diseases (21–33).

Ofengeim *et al.* showed the microglia of Alzheimer disease are dysfunctional and contain abundant pathological expression of RIPK1. Inhibition of Ripk1 kinase in a model of the disease reduced the burden of Aβ (amyloid beta) and pro-inflammatory cytokines, with improved memory. Taken as a whole, this work provides evidence to suggest that Ripk1 favours the development of a DAM state, in which the phagocytic capacity of microglia is impaired by abnormal activity of the endosomal/lysosomal pathway (34).

RIPK1 has been shown to regulate cell death and inflammation, and to be a driver of pathogenesis in inflammatory and degenerative disorders. This multifaceted molecule mediates cell signalling downstream of receptors such as TNFR1 (tumour necrosis factor receptor 1) and TLRs (toll like receptors). Whereas RIPK1 can act as a scaffold molecule to regulate pro-inflammatory and pro-survival responses essential for development and tissue homeostasis independent of its enzymatic activity, the kinase function is often required for initiating cell death associated with tissue inflammation: by activating caspase-8-dependent apoptosis, RIPK3 (receptor interacting protein kinase 3)/MLKL (mixed linage kinase like)-dependent necroptosis, and the highly inflammable, lytic form of cell death known as pyroptosis. With such powerful cytotoxic potential, it is likely that several mechanisms have evolved to control RIPK1 kinase activity (35,36). Post-translational modifications direct RIPK1 towards its scaffold function, including the NF-κB (nuclear transcription factor kappa B) protective pathway (37). A further means to inactivate RIPK1 is through proteolytic cleavage, specifically executed by caspase-8 which disables both scaffold and enzymatic functions (38,39). Autophosphorylation of RIPK1 is considered the principle target of its kinase activity; it is believed to induce conformational changes facilitating its association with cell death effectors (40,41).

To decouple the kinase-dependent and -independent functions of Ripk1, knocked-in mice have been generated; *Ripk1^D138N/D138N^* and *Ripk1^K45A/K45A^* mice by Polykratis *et al.* (42) and Berger *et al.* (43), respectively. The integrity of Ripk1 residues D138 and K45 is essential for RIPK1 catalytic activity, and disruption of either of these sites abrogates its enzymatic function, but its kinase-independent roles including the scaffold function are preserved. In contrast to the perinatal lethality of Ripk1-deficient mice, both kinase-dead mouse models are viable and develop normally, and thus Ripk1 kinase activity is not required for survival.

Vitner *et al.* reported upregulation of Ripk1 and Ripk3 in two murine models of the neuronopathic lysosomal disorder Gaucher disease. These changes occurred irrespective of whether the disease was induced genetically or chemically. Increased abundance of Ripk1 was also detected in a human Gaucher disease brain. When Gaucher disease was induced chemically in a mouse strain in which Ripk3 is ablated, the course of the neurological disease was attenuated with better motor function and greater survival. Upregulation of Ripk1 and Ripk3 was not observed in the brains of murine models of neuropathic sphingolipid diseases Niemann-Pick C1, GM1 and GM2 gangliosidosis (Sandhoff disease, SD). In contrast, the twitcher mouse brain displayed robust amounts of Ripk1 and Ripk3 (44).

Prompted by the remarkable improvement in neurological outcome after disruption of Ripk1/Ripk3 function in diseases of different aetiologies, we investigated the role of these and cognate molecules in the pathogenesis of Krabbe and in SD. Here, we also examined the expression of the signature DAM genes in both disease models and found their expression to be most prominent in macrophage/microglia with a phagocytic phenotype. As a first step towards understanding the relative Ripk1 functions in Krabbe disease, we intercrossed twitcher with the K45A kinase-dead Ripk1 mouse to homozygosity.

## Results

### Ripk1 expression differs between regions of the nervous system and murine models of Krabbe disease

**Figure 1:**
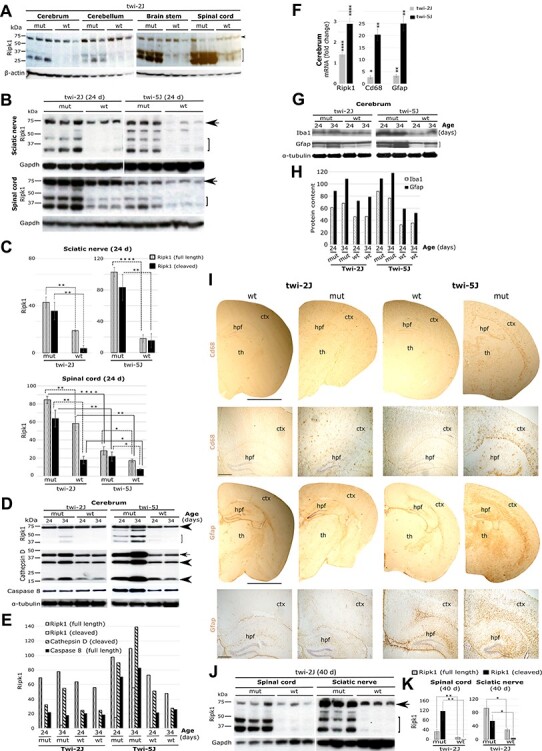
Spatiotemporal expression of Ripk1 in murine models of Krabbe disease. (**A**) Immunoblots of Ripk1 in different twi-2J CNS structures at HEP; arrowhead (full-length Ripk1), bracket (cleaved Ripk1); (**B**) Immunoblots of Ripk1 in sciatic nerve and spinal cord from twi-2J and -5J at P24; (**C**) Ripk1 densitometry of full-length and cleaved species relative to Gapdh in B; (**D**) Immunoblots of Ripk1, cathepsin D and caspase-8 (55 kDa, uncleaved) in twi-2J and -5J cerebrum at P24 and P34. Cathepsin D forms: arrow (intermediate), arrowheads (mature); (**E**) Densitometry of Ripk1, cathepsin D and caspase-8 relative to α-tubulin in D; (**F**) RT-qPCR of total RNA for *Ripk1* and gliosis markers *Cd68* and *Gfap* in twi-2J and -5J cerebrum at P24; (**G**) Immunoblots of Iba1 and Gfap in twi-2J and -5J cerebrum at P24 and P34; (**H**) Densitometry of Iba1 and Gfap relative to α-tubulin in G; (**I**) Bright field images of Cd68 IHC-stained cerebrum sections from twi-2J and -5J at their HEP, 40 and 24 days, respectively; (**J**) Immunoblots of Ripk1 in spinal cord and sciatic nerve in twi-2J at the HEP; (**K**) Densitometry of Ripk1 relative to Gapdh in J. For immunoblotting, 75–150 μg protein homogenate was loaded per lane. Blots were stripped of the first antibody and re-probed. Note, of the cleaved Ripk1 species only the lower form was used for densitometry as the upper one is often difficult to detect in wt animals. Cluster of differentiation 68 (*Cd68*), Glial fibrillary acidic protein (*Gfap*), Glyceraldehyde-3-phosphate-dehydrogenase (Gapdh), Receptor-interacting serine–threonine kinase 1(*Ripk1*), mutant (mut), wild type (wt), cerebral cortex (ctx), hippocampal formation (hpf) and thalamus (th). Scale bar: 2 mm for full size half cerebrums, and 200 μm for magnified views below. Student’s *t*-test; ^*^P ≤ 0.05; ^**^P ≤ 0.01; ^***^P ≤ 0.001; ^****^P ≤ 0.0001

The twitcher mouse (*Galc^twi-2J/twi-2J^*)—a genetically coherent murine model of disease—recapitulates neuropathological and biochemical features of acute infantile Krabbe disease. The cardinal pathological features of the disease remain essentially as first described by Knud Krabbe with loss of myelin and gliosis. It has been widely reported that cell loss in twi-2J nervous tissue occurs by apoptosis. This assertion is principally based on finding cells labelled with the TUNEL (terminal deoxynucleotidyl transferase dUTP nick end labelling) stain. However, necroptosis is another type of regulated cell death executed by Ripk1, Ripk3 and Mlkl. On finding over-representation of Ripk1 and Ripk3 molecules in the brain of twi-2J (44), we investigated their expression pattern. Ripk1 abundance was determined in cerebrum, cerebellum, brain stem and spinal cord at the humane end point (HEP) by immunoblotting. Our results corroborated findings by Vitner *et al.*, but abundance of Ripk1 in twitcher was not uniformly distributed between anatomical structures (Fig. 1A). As shown in this figure, expression of Ripk1 correlated with regions in which myelin degeneration and gliosis are most severe—findings compatible with involvement of Ripk1 in the pathogenesis of neuroinflammation.

We next compared expression of Ripk1 in two distinct murine models of Krabbe disease. The twi-2J, the most intensely studied and widely used experimentally, is caused by an *opal* nonsense mutation in the *Galc* gene and no Galc protein is detectable (16). A more recently characterised mutation occurs in the twi-5J mouse (45); this harbours a missense mutation that inactivates Galc catalytic activity without the loss of protein expression. Apart from differences in disease-causing mutations and the absence or presence of Galc immunoreactivity, it should be pointed out that these models differ in their genetic backgrounds, C57BL6/6J and BXD32/TyJ for twi-2J and twi-5J, respectively. BXD32/TyJ is an inbred strain resulting from interbreeding C57BL/6J and DBA/2J mice. Both models show neuropathological features of krabbe disease, but twi-5J is the most severe: less than one in four mutant twi-5J remain alive at age P24, whereas almost all twi-2J survive to P35. According to Potter *et al.*, in twi-5J there appears to be no correlation between the toxic metabolite psychosine and demyelination or axonal loss, which at first glance militates against the predictive power of psychosine as a prognostic indicator for the acute disease as appear to be the case in the twi-2J strain. With potential clinical applications in mind that might be uncovered by exploring disease mechanisms in these strains, we investigated whether these divergent phenotypes might associate with differences in Ripk1 expression.

Ripk1 was quantified by immunoblotting in sciatic nerve and spinal cord in twi-2J and twi-5J at P24—the approximate HEP for twi-5J mice. The abundance of immunoreactive full-length Ripk1 in sciatic nerve was slightly greater in mutant twi-5J than in twi-2J, but for the cleaved forms the changes were not statistically significant (Fig. 1B and C). In the spinal cord, more full-length and cleaved forms of Ripk1 were detected in twi-2J than twi-5J (Fig. 1B and C). However, this was true for both mutant and wild type controls, suggesting intrinsic strain differences in Ripk1 expression. Nevertheless, the ratio of full-length to cleaved Ripk1 species did not differ materially between the strains.

At P25, myelin is preserved in the cerebral cortex of twi-2J but, Potter *et al.* showed that cortical myelin was lost at this age in twi-5J (45). Based on our earlier findings that Ripk1 abundance appears to correlate with demyelination and gliosis, we predicted that more Ripk1 would be present in the cortex of twi-5J than in twi-2J mice. In agreement with survival data from Potter *et al.*, a few of our twi-5J mice lived beyond 30 days, thereby approaching the natural HEP of the twi-2J strain. Taking advantage of the availability of these older twi-5J mice, we examined expression of Ripk1 in the cerebrum of the twitcher strains at P24 and P34. This showed that the abundance of Ripk1 increased with age (Fig. 1D and E), and that the cerebrum of twi-5J expressed more Ripk1; it was readily detectable at P24 in twi-5J, but absent in twi-2J at this age. As can be seen in Figure 1D and E, the amount of Ripk1 in the cerebrum of twi-5J at P24 exceeded the quantity detected at P34 in twi-2J.

To further define the relationship between Ripk1 expression and disease severity in the cerebrum of the twitcher strains, we examined cathepsin D, a well-known marker of disease. We found increased cathepsin D immunostaining, concordant with the altered abundance of Ripk1 (Fig. 1D and E). Furthermore, caspase-8 was similarly changed in the cerebrum of twi-5J, but not in twi-2J (Fig. 1D and E). Transcriptional abundance of Ripk1, and inflammatory markers Cd68 (Cluster of differentiation 68) and Gfap (Glial fibrillary acidic protein) was assessed in the cerebrums of both strains at P24 by real-time (RT) quantitative PCR (RT-qPCR). Although expression of these transcripts was significantly upregulated in all mutant animals, it was greater in twi-5J (Fig. 1F). Evaluation of the inflammatory response at the translational level by immunoblotting (Fig. 1G and H) and immunohistochemical staining (IHC; Fig. 1I) mirrored the RT-qPCR results. We should mention here that we favoured immunoblotting against Cd68 for direct comparison. However, our antibody which clearly stains tissue sections by IHC, does not appear to work when used in this application.

To follow changes in Ripk1 expression during the longer lifespan of twi-2J, we examined spinal cord and sciatic nerve at P40, and found differential expression at these sites (Fig. 1J and K). In the spinal cord, the cleaved and therefore inactive species of Ripk1 was most abundant, but in the sciatic nerve the full-length, and potentially functionally active 74 kDa species predominated.

Overall the findings show that expression of Ripk1 associates closely with the severity and progression of demyelination and gliosis in Krabbe disease, modelled in the twitcher strains. However, these studies cannot be expected to demonstrate a causal role for Ripk1 in pathogenesis, nor provide a mechanistic explanation for phenotypic differences between the two twitcher strains that can be attributed to Ripk1 expression. Rather, given the decisive contribution of Ripk1 to the molecular pathogenesis of several other diseases, the current findings clearly implicate an active role for this central mediator of cell death and inflammation in the florid neuropathology of Krabbe disease.

### Molecular partners engaged in Ripk1 signalling are upregulated in Krabbe and SD

The spinal cord serves as a paradigmatic site of neurodegeneration during the evolution of Krabbe disease and as a structure that is myelinated early in development, it is correspondingly one of the first to undergo demyelination. To determine whether additional molecules known to be connected with Ripk1 signalling might be overrepresented in twi-2J, we used RT-qPCR of total ribonucleic acid (RNA) to define expression at three key stages of disease progression: (1) 11 days (P11; pre-symptomatic), (2) 20 days (P20; early symptomatic) and (3) HEP 39–42 days.

**Figure 2:**
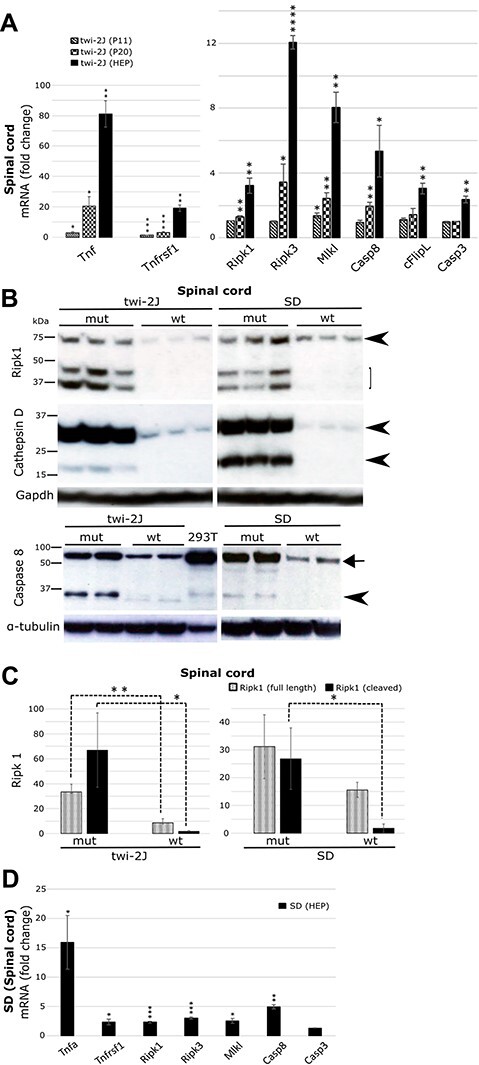
Core elements of the ripoptossome/necroptosome are upregulated in mouse models of Krabbe and SD. Spinal cord was examined as a paradigm of disease. (**A**) RT-qPCR of total RNA at three stages of disease in twi-2J with markers *Tnf*, *Tnfrsf1*, *Ripk1, Ripk3, Mlkl, Casp8, cFlipL* and *Casp3*; (**B**) Immunoblots of Ripk1, cathepsin D and caspase-8 in twi-2J and SD mice at the HEP. 293T cells transfected with a plasmid expressing murine caspase-8 was used as a positive control for the antibody against caspase-8. One hundred microgram protein homogenate was loaded per lane. Blots were stripped of first antibody and re-probed; (**C**) Densitometry of Ripk1 relative to α-tubulin in B. Note, of the cleaved Ripk1 species only the lower one was used for densitometry as the upper form is often difficult to detect in wt animals. (**D**) RT-qPCR of total RNA from SD at HEP with markers: *Tnfrsf1*, *Ripk1, Ripk3, Mlkl, Casp8 and Casp3*. Tumour necrosis factor (*Tnf*), Tumour necrosis factor receptor 1 (*Tnfrsf1),* Receptor-interacting serine-threonine kinase 1(Ripk1), Receptor-interacting serine-threonine kinase 3 (Ripk3), Mixed lineage kinase domain-like (*Mlkl*), Caspase-8 (*Casp8*), Caspase-3 (*Casp3*). mutant (mut), wild type (wt). Student’s *t*-test; ^*^P ≤ 0.05; ^*^^*^P ≤ 0.01; ^*^^*^^*^P ≤ 0.001; ^*^^*^^*^^*^P ≤ 0.0001.

Expression of the cytokine tumour necrosis factor (*Tnf*) is known to be increased in Krabbe disease: by P11 the abundance of *Tnf* RNA was already elevated in the spinal cord and expression of the cognate receptor, *Tnfrsf1*, mirrored this alteration (Fig. 2A). Compared with spinal cord from control mice, transcription of *Ripk1*, *Ripk3*, *Mlkl* and *Casp8* also increased progressively with advancing age. In contrast, *Casp3* was significantly raised only at the HEP (Fig. 2A). *Fadd* (Fas-associated death domain protein) whose role in Tnf-induced apoptosis is the recruitment of caspase-8 was increased by 2-fold (*P* ≤ 0.05) in twi-2J at the HEP, the only time studied. The Fas receptor encoded by *Fas*, belongs to the same family of death receptors as Tnfr1, and transduces death signals after engagement by FasL (Fas ligand). This in turn leads to the recruitment first of Fadd, followed by caspase-8, which after activation by homodimerization, initiates the apoptotic cascade. *Fas* expression was explored only at the HEP, and was upregulated 6-fold in twi-2J (*P* ≤ 0.001).

Transcription factor NF-κB drives the expression of pro-survival gene cFlipL [cellular caspase-8 (FLICE)-like inhibitory protein, long isoform], which heterodimerizes with caspase-8 and inhibits its activation, abolishing apoptosis. Expression of *cFlip_L_* RNA increased in the spinal cord over time (Fig. 2A).

Findings by Vitner and colleagues suggested that Rip1 and Ripk3 expression was selectively increased in Gaucher and Krabbe disease brain, and did not report such changes in experimental models of other sphingolipidoses, such as Niemann-Pick C or the GM2 gangliosidosis SD. To explore comparatively the pathology of these diseases, and initially intended as a source of control tissue, we also examined expression of Ripk1 in SD mice. Ripk1 expression in spinal cord was quantified by immunoblotting in twi-2J and SD mice at their respective HEPs, 40 days and 4 months. Our results differed from those previously reported, Ripk1 was clearly upregulated in both diseases (Fig. 2B and C). Cathepsin D and caspase-8 were similarly over-represented in both models of disease (Fig. 2B). To evaluate the potential involvement of components of the Ripk1 signalling cascades in SD, transcripts were quantified by RT-qPCR in spinal cord, and showed that *Tnf*, *Tnfrsf1*, *Ripk1*, *Ripk3*, *Mlkl* and *Casp8* were increased, albeit to a lesser extent than in twi-2J at the HEP. No difference in the amounts of *Casp3* was seen in SD (Fig. 2D).

In summary, Ripk1 is abnormally expressed in nervous tissue as the severity of disease increases with age in twitcher mice; several cognate molecules previously known to be involved in mechanisms of disease, inflammation and cell death, were also progressively upregulated. Abnormal expression of these molecules was likewise identified in the mouse model of SD, indicating that Ripk1-related processes are not unique to Gaucher and Krabbe diseases and are more likely shared with other neurodegenerative sphingolipidoses. Nevertheless, whether these molecules are of primary biological importance to the disease process, their functional role remains to be elucidated.

### The gene expression signature of disease-associated microglia and neuroinflammation are prominent features in Krabbe and SD

**Figure 3:**
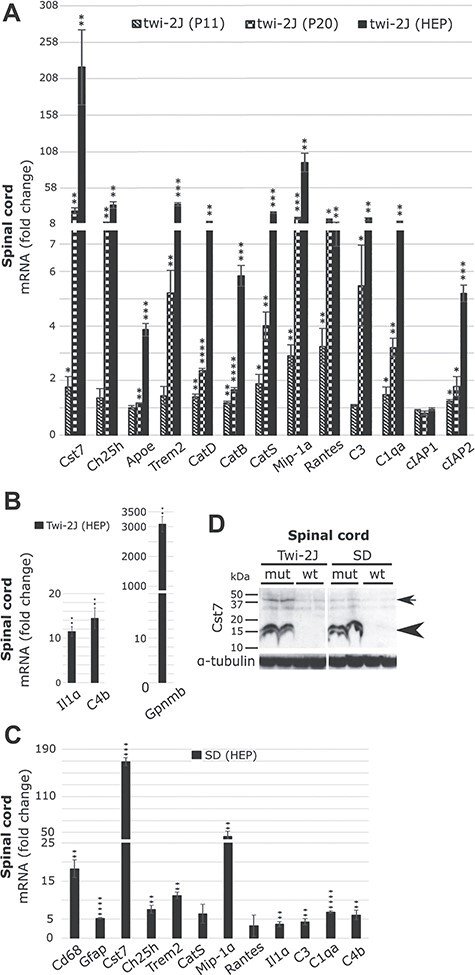
Upregulated expression of DAM and neuroinflammation genes is a feature of Krabbe and SD. Spinal cord was examined as a paradigm of disease. (**A**) RT-qPCR of total RNA at three stages of disease was studied in twi-2J for *Cst7*, *Ch25h*, *Apoe*, *Trem2*, *CatD*, *CatB*, *CatS*, *Mip-1α*, *Rantes*, *C3*, *C1qa*, *cIAP1* and *cIAP2*; (**B**) RT-qPCR of total RNA of *Il1α*, *Cd4* and Gpnmb in twi-2J at HEP; (**C**) RT-qPCR of total RNA for the above markers in SD at HEP; (**D**) Immunoblot of Cst7 in twi-2J and SD at their respective HEP. Two hundred microgram protein homogenate was loaded per lane. Blots were stripped of first antibody and re-probed with anti-α-tubulin; Cst7 dimers (arrow) and monomers (arrowhead). Cystatin F *(Cst7)*, Cholesterol 25-hydroxylase *(Ch25h)*, Apolipoprotein E *(Apoe)*, Triggering receptor expressed on myeloid cells 2 (*Trem2)*, Cathepsin D (*CatD)*, B (*CatB)*, S (*CatS)*, Macrophage inflammatory protein 1 alpha *(Mip-1α)*, Regulated on activation normal T cell expressed and secreted (*Rantes)*, Complement component 3 (*C3)* and component 1, q subcomponent *(C1qa)*, Cellular Inhibitor of Apoptosis Protein 1 (*cIAP1)* and 2 (*cIAP2),* Interleukin 1 alpha *(Il1α)*, Complement component 4B *(Cd4)* and Glycoprotein non-metastatic melanoma protein B (*Gpnmb*). mutant (mut), wild type (wt). Student’s *t*-test; ^*^P ≤ 0.05; ^*^^*^P ≤ 0.01; ^*^^*^^*^P ≤ 0.001; ^*^^*^^*^^*^P ≤ 0.0001.

Findings by Ofengeim *et al.* suggested that Ripk1 regulates the expression of DAM genes, including *Cst7* (Cystatin F) and *Ch25h* (Cholesterol 25-hydroxylase) in Alzheimer disease (34). In light of this, we examined the temporal expression of these genes in Krabbe disease. We analyzed twi-2J RNA from spinal cord at P11, P20 and HEP by RT-qPCR: *Cst7* and *Ch25h* are highly upregulated, and transcript abundance increases with disease progression (Fig. 3A). *Cst7* is elevated above control samples as early as P11. To determine whether the upregulation of *Cst7* might be a response to heighten cathepsin activity, we examined *CatB*, *D* and *S*, and found that expression of all three proteases is increased progressively, and significantly so from P11, the earliest time point studied.

Important players in the DAM response *Apoe* (Apolipoprotein E) and *Trem2* (Triggering receptor expressed on myeloid cells 2) were also quantified by RT-qPCR in twi-2J RNA from spinal cord: they were upregulated at P20 and HEP, but not at P11 (Fig. 3A). This concurs with previous work indicating that these DAM genes are expressed at relatively late stages of disease (21). More recently, *Gpnmb* (Glycoprotein non-metastatic melanoma protein B) was shown to be also upregulated in DAM (22), and in twi-2J, we found its expression increased several 1000-fold above control levels at the HEP, the only time examined (Fig. 3B).

The suggested role of Ripk1 in inflammatory responses downstream of Tnf stimulation and signalling is based on strong foundations. Stimulation of cells with Tnf induces the expression of cytokines *Mip-1α* (macrophage inflammatory protein 1 alpha) and *Rantes* (regulated on activation normal T cell expressed and secreted), also known as *Ccl3* and *Ccl5*, respectively. To understand the possible engagement of Ripk1 in inflammatory responses associated with Krabbe disease, we evaluated the dynamic expression of these cytokines in the spinal cord of twi-2J by RT-qPCR at three distinct stages of disease. As seen in Figure 3A, *Mip-1α* and *Rantes* transcripts are progressively upregulated in twitcher (Fig. 3A).

Cellular inhibitors of apoptosis proteins 1 and 2 (cIAP1 and cIAP2) belong to a family of inhibitors that de-escalate the commitment of cells to die, they do this by ubiquitinating molecules such as Ripk1 that might otherwise favour cell death over survival, and by marking specific substrates for proteosomal degradation. These paralogous proteins can be differentially expressed, suggesting they have distinct, non-redundant functions. There is evidence that transcription of cIAP2 is controlled by NF-κB, and its expression has been noted in the polarised pro-inflammatory defensive monocyte/macrophage M1 phenotype. In contrast, cIAP1 is preferentially expressed in the anti-inflammatory tissue-repair M2 phenotype (46). Despite the fact that the origins of macrophage and microglia are distinct, they share cellular functions and markers that change according to their polarization state. It has been suggested that their functions in phagocytosis and regulation of inflammation are controlled by Trem2 (47). Because cIAP1/2 are also at the early junction between cell survival and death, and intimately connected to Ripk1 signalling, we examined their transcriptional expression in the spinal cord of twi-2J during the course of the disease. Results were unambiguous: cIAP1 expression in the spinal cord of mutant twitcher remained unchanged compared with healthy controls, but upregulation of cIAP2 was apparent from P11, and increased over time (Fig. 3A). This suggests that activated macrophage/microglia in Krabbe disease are of the pro-inflammatory M1 phenotype.

Activation of complement occurs in many acute and chronic neurodegenerative disorders, but it is unknown whether complement contributes to the pathogenesis of Krabbe disease. To investigate this, we determined transcript abundance of complement components *C3*, *C1qa* and *C4b* in the spinal cord of twi-2J by RT-qPCR. All three genes were overexpressed, and expression of the alternative pathway, *C3*, and classical pathway, *C1qa*, components increased markedly with age (Fig. 3A). Liddelow *et al.* established that the activation of microglia and astroglia during a neurodegenerative process does not occur independently; instead, microglia induces activation of astroglia by secreting Il-1α, Tnfα and C1q; they showed, the combination of these cytokines is necessary and sufficient to activate astrocytes. The homeostatic function of astrocytes is thus altered, and they are turned into effectors of death to neurons and oligodendrocytes (48). Having observed transcriptional upregulation of *Tnf* and *C1qa* in twitcher, we examined *Il-1α* (Interleukin 1 alpha) expression at the HEP and founded it to be upregulated (Fig. 3B).

The findings by Ofengeim *et al.* in Alzheimer’s disease and DAM gene signature reported here in Krabbe disease, led us to consider the inflammatory response in murine SD. In agreement with previous reports, enhanced transcriptional activation of micro- and astroglia with cell type-specific markers *Cd68* and *Gfap*, in the spinal cord at the HEP by RT-qPCR was confirmed (Fig. 3C). Expression of *Cst7*, *Ch25h*, *Trem2*, *CatS*, *Mip-1α*, *Rantes*, the complement components, *C3*, *C1qa* and *C4b* and *Il-1α* was examined, and except for *CatS* and *Rantes*, all were significantly upregulated (Fig. 3C). In reporting these parallel findings in SD, it is important to note that expression of most markers was more elevated at the HEP in twitcher, compared with Sandhoff mouse tissues. Given the putative role of *Cst7* in the neurodegenerative process that characterizes these sphingolipidoses, we also investigated expression of the Cst7 protein by immunoblotting: in the spinal cord cytostatin F was strongly and similarly overrepresented in both murine models (Fig. 3D). We conclude that despite the divergent genetic basis and cytopathological features of these diseases, elements of the inflammatory response and the recently characterised microglia disease entity DAM, are shared between these two sphingolipidoses—and indeed with changes reported in Alzheimer’s disease and other neurodegenerative disorders.

### Autophagosomal flux and function is impaired in the twitcher and Sandhoff mouse

Ofengeim *et al.* proposed and provided tentative evidence that a dysfunctional lysosome or ubiquitin/proteosomal system (UPS) leads to the induction of Ripk1 enzymatic activity, which in term causes an altered microglia state with upregulation of genes such as Ch25h and Cst7. Cst7-transfected cells promoted the accumulation of p62 and LC3II (34).

**Figure 4:**
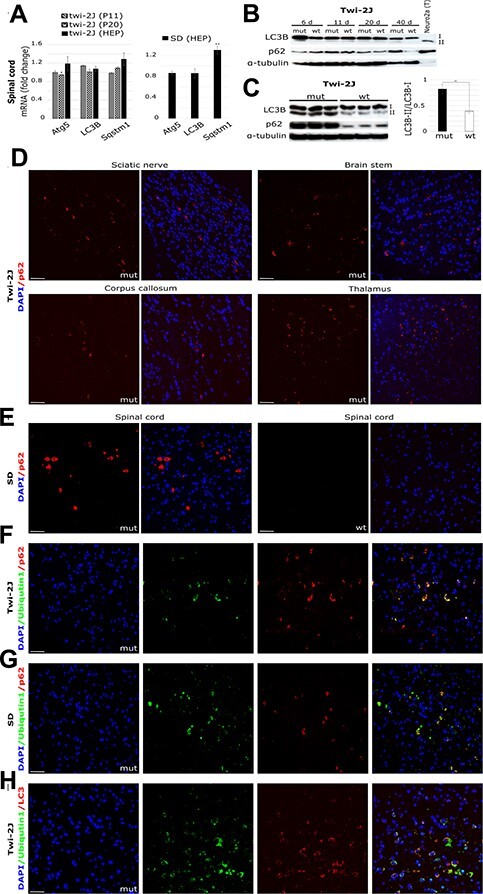
Autophagosomal/lysosomal dysfunction accompanies Ripk1 expression in Krabbe and SD. Autophagy was studied by (**A**) RT-qPCR of total RNA in spinal cord at three stages of disease in twi-2J and at the HEP in SD, with markers *Atg5* (Autophagy related 5), *LC3B* (Microtubule-associated protein 1 light chain 3 beta), and *Sqstm1* (Sequestosome 1 codes for p62); (**B**) Immunoblot of LC3B and p62 in twi-2J spinal cord at different ages. Protein extracts from Neuro2a chloroquine-treated cells were used as positive control of upregulated autophagy; (**C**) Immunoblot to LC3B and p62 and densitometry in twi-2J brain stem at HEP. Seventy-five microgram protein homogenate was loaded per lane. Blots were stripped of first antibody and re-probed with subsequent antibodies. Fluorescent IHC staining of p62 in twi-2J sciatic nerve and brain (**D**), and SD spinal cord at HEP (**E**). Dual IHC staining of ubiquitin1 and p62 in twi-2J spinal cord at HEP (**F**) and SD (**G**). (**H**) Dual IHC staining of ubiquitin1 and LC3B in twi-2J spinal cord at HEP. Nuclear stain: DAPI (4′,6-Diamidine-2′-phenylindole dihydrochloride), mutant (mut), wild type (wt). Student’s *t*-test; ^*^P ≤ 0.05; ^*^^*^P ≤ 0.01.

Having established that Ch25h and Cst7 are overrepresented in twitcher and Sandhoff mice, we searched for perturbation in autophagosomal/lysosomal function. Transcriptional expression of *Atg5*, *LC3B* and *Sqstm1* (coding for protein p62), key molecules in UPS/autophagosome function, during disease progression in the spinal cord of twi-2J by RT-qPCR was not clearly changed at any stage, but there was an upwards trend for *Sqstm1*, without reaching statistical significance (Fig. 4A). Expression of *Sqstm1* was slightly upregulated in the spinal cord of the Sandhoff mouse at the HEP (Fig. 4A). LC3B and p62 protein abundance at P6, P11, P20 and P40 in twitcher, showed only differential expression at the HEP (age 40 days; Fig. 4B). To corroborate the result in a different structure, we studied the brain stem at this age and found substantial increases in p62 and LC3B; the LC3B-II/LC3B-I ratio was 2-fold greater in mutant animals compared with healthy controls (Fig. 4C). IHC staining of sciatic nerve and brain sections with antibodies against p62 revealed discrete labelling of a granular appearance, not only in myelinated regions but also in grey matter (Fig. 4D). Abnormal staining of p62 was present in the brains and nerves at earlier stages of disease, P11 and P24, albeit less abundantly (data not shown). The patter of p62 IHC staining in the SD brain localised to areas of grey matter, and no staining was observed in wild type counterparts of either strain (Fig. 4E).

To evaluate whether abnormalities in autophagosomal function might be linked to alterations in the UPS system, we used double IHC staining with antibodies against p62 and ubiquitin1 on twi-2J and SD mouse sections at the HEP. Most p62-positive cells also stained with ubiquitin1 (Fig. 4F and G). Moreover, dual labelling with ubiquitin1 and LC3 demonstrated frequent co-localization of these markers (Fig. 4H).

**Figure 5:**
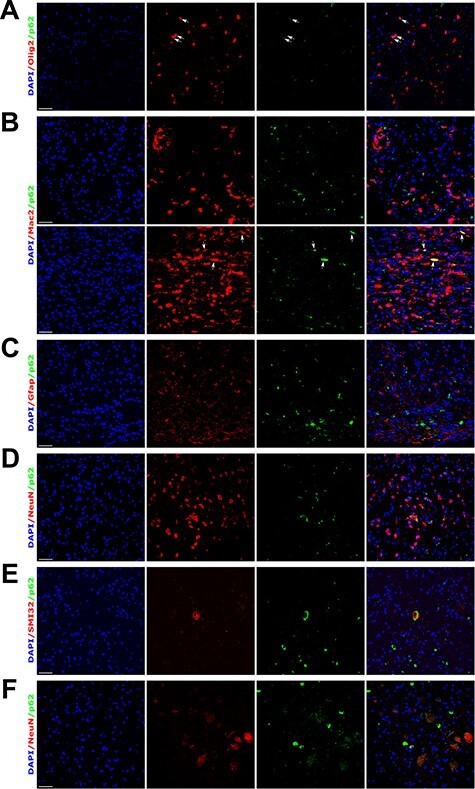
p62 accumulates principally in oligodendroglia and neurones in twi-2J and in neurones in SD mice. (**A-E**) Dual IHC staining of mutant twi-2J at HEP with: p62 and oligodendroglia Olig2 (**A**), p62 and macrophage/microglia Mac2 (**B**), p62 and astroglia Gfap (**C**), p62 and neuronal NeuN (**D**), p62 and non-phosphorylated neurofilament protein SMI32 (**E**); and p62 and neuronal NeuN in SD (**F**). All sections are from spinal cord, except in B where sciatic nerve (bottom panels) is also shown. Nuclear stain: DAPI (4′,6-Diamidine-2′-phenylindole dihydrochloride. Scale bars: 50 μm.

To identify the cell types with abnormal p62 expression, we co-stained with cell type-specific markers. In twi-2J, some p62-positive cells co-localised with Olig2 (oligodendroglia transcription factor Olig2; Fig. 5A). There was occasional co-localized staining with the microglial phagocytic marker Mac2 (Fig. 5B), which we attribute to phagocytosis of p62-containing cellular debris by macrophage/microglia. We were unable to identify Gfap-stained cells that were also positive for p62 (Fig. 5C). In contrast, some NeuN-positive cells had a p62 punctate pattern of expression, which was found throughout the neuraxis (Fig. 5D). To establish whether these NeuN-positive cells might be degenerating neurons, we used antibody SMI32 (specific for non-phosphorylated neurofilament protein), and identified SMI32-stained axons and neuronal cell bodies co-labelled with p62, which had a ring-like perinuclear distribution (Fig. 5E). In the Sandhoff brain, p62 often co-localised with NeuN (Fig. 5F), but not with Olig2, Mac2 or Gfap (data not shown). In summary, p62 appears to be abnormally expressed preferentially in oligodendroglia and degenerating neurones in twitcher, and in neurones in SD mice.

The discrete cellular accumulation of p62/ubiquitin1/LC3 in what appears as organelle-like structures is compatible with thwarted autophagosome function that has not been trafficked to completion, a defect in autophagic flux. Although dysregulation of the UPS, autophagosomal and lysosomal systems is to be expected in lysosomal diseases, Alzheimer and other diseases are not primarily caused by defects in the lysosome, indicating that there is very little specificity in the activation of the pathological cascades.

### Ripk1 localizes to macrophage/microglia with an activated/phagocytic phenotype

It is important to identify the tissue distribution and the cell types that overexpress Ripk1 as this may suggest potential pathological mechanisms. Ripk1-specific labelling was not detected after standard IHC protocols although antibodies were successful in immunoblotting studies. However, the combined use of a mouse-specific mRNA *in situ* hybridization (ISH) Ripk1 probe (Mm-Ripk1-C1 # 464511, RNAscope technology, Bio-Techne) and IHC with cell-type specific markers proved fruitful (49).

**Figure 6:**
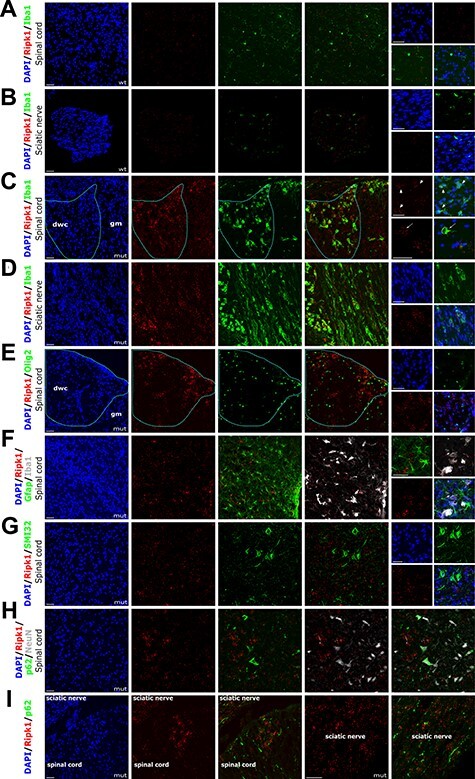
Ripk1 localizes to activated macrophage/microglia. (**A-I**) Combined ISH with RNAscope probe Mm-Ripk1-C1 and fluorescent IHC staining of twi-2J with cell type-specific markers for macrophage/microglia Iba1(**A**-**D**), oligodendroglia Olig2 (**E**), macrophage/microglia Iba1 and astroglia Gfap (**F**), non-phosphorylated neurofilament protein SMI32 (**G**), p62 and neuronal marker NeuN (**H**), and p62 (**I**). Nuclear stain: DAPI (4′,6-Diamidine-2′-phenylindole dihydrochloride. Mutant (mut), wild type (wt), dorsal white column (dwc), grey matter (gm). Scale bars: 50 μm.

To establish the specificity of probes and test the technique and quality of our tissue sections, we used negative and positive control probes, # 320871 (to bacterial *DapB*) and # 320881 (a 3-plex probe mix to mouse *Polr2a*-C1, *Ppib*-C2 and *Ubc*-C3), respectively (results not shown). ISH with the mRNA Ripk1-C1 probe was followed by IHC staining for the microglia marker Iba1 on spinal cord and sciatic nerve sections from Twi-2J (age 40 days). Ripk1 was expressed at low levels in wild type tissues, and as expected, Iba1-stained cells had a ramified morphology, typical of non-activated macrophage/microglia, and minimal Ripk1 signal (Fig. 6A and B). In contrast, mRNA Ripk1 signal congregated on Iba1-labelled cells in mutant sciatic nerve and it was also prominent in white matter in spinal cord, such as the dorsal column that is affected by demyelination. These Iba1-positive cells tended to cluster, and had a globoid morphology with reduced ramification, characteristic of reactive macrophage/microglia. The Ripk1 mRNA staining was detected in the nucleus, cytoplasm and cell processes (Fig. 6C and D). A similar staining pattern was observed when an antibody against Mac2 was used instead of Iba1 (data not shown). Little co-localization occurred on labelling with the Ripk1 probe and antibody to Olig2 (Fig. 6E). Because astrogliosis and microgliosis are connected and these cells phagocytose degenerating myelin, to ascertain Ripk1 macrophage/microglia-specific labelling, we simultaneously applied the Ripk1 probe and antibodies to Gfap and Iba1 to spinal cord and brain sections. Little Ripk1 signal was found in astrocytes located in close proximity to Iba1 cells that were heavily stained with Ripk1 (Fig. 6F). Co-staining sections with the Ripk1 probe and the antibody to non-phosphorylated neurofilament protein SMI32 failed to show co-localization (Fig. 6G). Probing with the Ripk1 probe and antibodies to NeuN and p62, again demonstrated NeuN-positive cells labelled with p62, but there was little staining of these cells with the Ripk1 probe, although heavy staining of suspected reactive macrophage/microglia occurred in close proximity (Fig. 6H). Careful examination of sciatic nerve and spinal cord for Ripk1 and p62 co-localization failed to show any association, and similar studies in Sandhoff mouse spinal cord sections had less Ripk1 staining, and only macrophage/microglia with an activated morphology expressed appreciable quantities of Ripk1 (Supplementary Material, Fig. S1).

We conclude that as reported in microglia in the APP/PS1 (amyloid precursor protein/presenilin 1) murine model of Alzheimer’s disease (34), upregulation of Ripk1 in both twitcher and SD mice, predominantly occurs in activated macrophage/microglia.

### Genetic ablation of Ripk1 kinase activity in twitcher does not alter disease progression or survival nor neuropathological features

**Figure 7:**
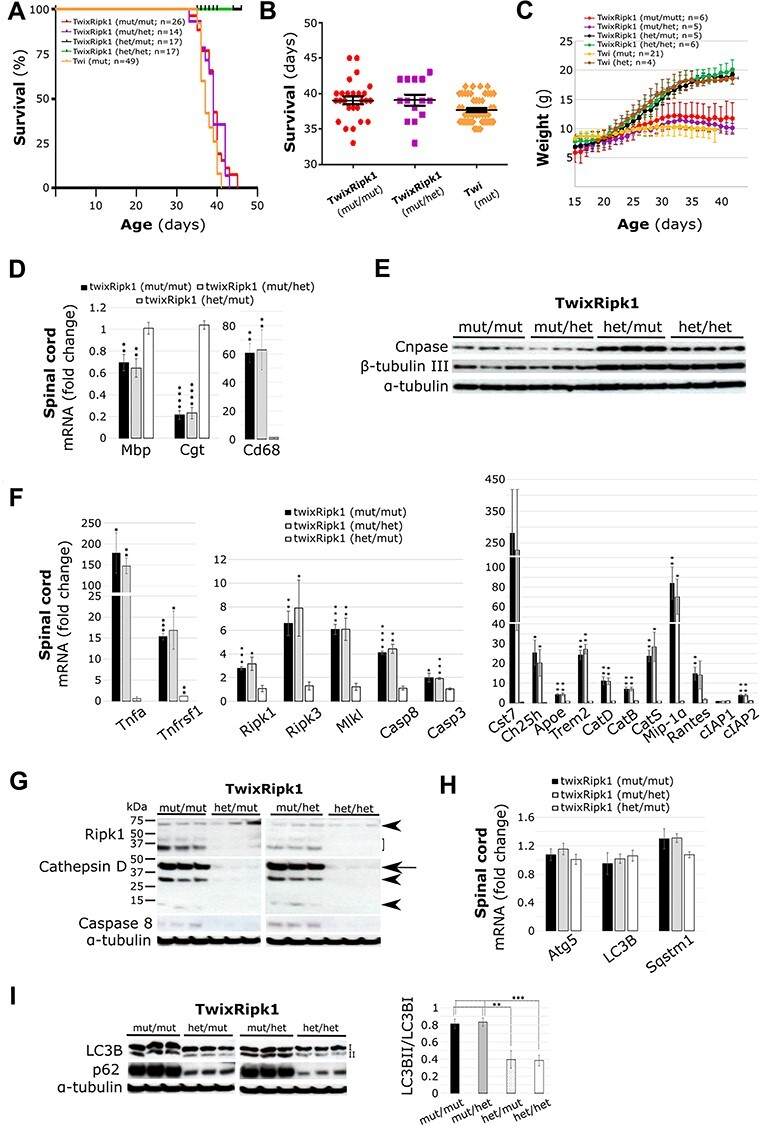
Genetic inhibition of Ripk1 kinase activity neither extends life nor prevents disease progression or pathological features in twitcher. (**A**, **B**) Survival of mutant twi-2J (twi) which is wild type for *Ripk1* does not differ from twi homozygous or heterozygous for the K45A Ripk1 mutation, and thus, no survival benefit was accrued. Kaplan–Meier (**A**) and scatter plot with mean ± SEM (**B**). (**C**) Mice were weighed daily from age 15 days until they reached the HEP and mean ± SD was plotted. The K45A Ripk1 mutation whether in homo- or heterozygosity does not alter the weight of heterozygous or mutant twitchers. (**D**) Myelin defects and gliosis was assessed by RT-qPCR of total RNA in the spinal cord HEP with markers: Mbp (Myelin basic protein), *Cgt* (UDP-galactose:ceramide galactosyl-transferase), and *Cd68* (Cluster of differentiation 68); (**E**) Myelin and axon protein content was examined in sciatic nerve with markers Cnpase (2′,3’-Cyclic Nucleotide 3’-Phosphodiesterase) and β-tubulin III, respectively by immunoblotting at HEP. (**F**) Evaluation of expression of core ripoptosome/necroptosome components, neuroinflammation and DAM genes was performed by RT-qPCR of total RNA from HEP spinal cord with: Tumour necrosis factor (*Tnf)*, Tumour necrosis factor receptor (*Tnfrsf1)*, Receptor-interacting serine–threonine kinase 1 (*Ripk1),* Receptor-interacting serine–threonine kinase 3 *(Ripk3),* Lineage kinase domain-like *(Mlkl), Caspase-8 (Casp8),* Caspase-3 (*Casp3)*; Cystatin F *(Cst7)*, Cholesterol 25-hydroxylase *(Ch25h)*, Apolipoprotein E *(Apoe)*, Triggering receptor expressed on myeloid cells 2 (*Trem2)*, Cathepsin D (*CatD)*, B (*CatB)*, S (*CatS)*, Macrophage inflammatory protein 1 alpha *(Mip-1α)*, Regulated on activation normal T cell expressed and secreted (*Rantes)*, Cellular Inhibitor of Apoptosis Protein 1 (*cIAP1)* and 2 (*cIAP2).* (**G**) Immunoblotting of Ripk1, cathepsin D and caspase 8 from brain stem at HEP. Ripk1 species full-length (arrowhead), cleaved forms (bracket); catepsin D intermediate (arrow) and mature forms (arrowhead); caspase-8 (uncleaved). Autopohagy was examined by (**H**) RT-qPCR of total RNA from HEP spinal cord with markers *Atg5* (autophagy related 5), *LC3B* (microtubule-associated protein 1 light chain 3 beta), and *Sqstm1* (sequestosome 1 codes for p62); (**I**) Immunoblotting of brain stem with LC3BII and p62, and ratio LC3BII/LC3B1 relative to α-tubulin. RT-qPCR RNA quantification is given relative to healthy controls (TwixRipk1: het/het), matched for age and sex. Ten microgram protein homogenate was loaded per lane in B, and 100 μg for D and F. Blots were stripped of first antibody, and re-re-probed with subsequent antibodies. Mutant (mut), heterozygote (het). Student’s *t*-test; ^*^P ≤ 0.05; ^*^^*^P ≤ 0.01; ^*^^*^^*^P ≤ 0.001; ^*^^*^^*^^*^P ≤ 0.0001.

Unlike total Ripk1-deficient mice which die perinatally, the kinase-dead knock-in *Ripk1^K45A/K45A^* (42) and *Ripk1^D138N/D138N^* (43) mice develop normally. To test the pathological role of Ripk1 kinase activity definitively in a Krabbe disease model, we crossed heterozygous twi-2J (*Galc^twi-2J/+^*) with *Ripk1^K45A/K45A^* mice to homozygosity. Survival was not influenced by the sex of the animals and data from both sexes was pooled and analyzed with the log-rank (Mantel–Cox) test, and visualised as Kaplan–Meier and scatter plots (Fig. 7A and B). Survival of the different genetic crosses was examined by one-way analysis of variance (ANOVA) and adjusted for multiple post-hoc comparisons by the Bonferroni method. Homozygous mutant Twi-2J (*Galc^twi-2J/twi-2J^*) mice mean survival in days (± standard error of the mean [SEM]) were respectively 37.6 ± 0.27 (*n* = 49), 39 ± 0.56 (*n* = 26) for *Galc^twi-2J/twi-2J^ Ripk1^K45A/K45A^* and 39 ± 0.78 (*n* = 14) for *Galc^twi-2J/twi-2J^ Ripk1^K45A/+^*; with no significant difference between the genotypes (*P* > 0.05; Fig. 7B). Thus, there was no survival benefit when mutant twi-2J (*Galc^twi-2J/twi-2J^*) was either homozygous or heterozygous for the *Ripk1* kinase-dead K45A mutation, (*Galc^twi-2J/twi-2J^ Ripk1^K45A/K45A^*) and (*Galc^twi-2J/twi-2J^ Ripk1^K45A/+^*), respectively.

Body weight, a general indicator of wellbeing in mice, was recorded daily from age 15 days to the HEP in small cohorts of females. There were no distinguishable differences between the genotypes up to the age of about 20 days, but soon after, a clear split between homozygous and heterozygous mice for the Galc mutation (*Galc^twi-2J^*) was noticed, and at the terminal stages of the disease all *Galc^twi-2J/twi-2J^* mice lost weight and required humane killing. No difference in growth was observed between heterozygous twi-2J that were wild type, heterozygous or homozygous for the K45A *Ripk1* mutation. Trend analysis showed that whereas mice wild type for *Ripk1* lost more weight over the course of the disease than those homozygous for the K45A *Ripk1* mutation, the weight of heterozygotes was intermediate (Fig. 7C). Also, worth noting was the difference in the survival trend which was the lowest for homozygous mutant twi-2J that were wild type for *Ripk1*. We attribute these small differences to the highly inbred nature of our twi-2J mouse colony.

We investigated whether ablation of Ripk1 kinase activity caused subtle changes in pathology at the molecular level in twitcher, which although not extending life might induce cryptic benefit. We studied structures from the same animals by different techniques. We first analyzed the transcriptional expression of the myelin/oligodendrocyte markers *Mbp* (myelin basic protein) and *Cgt* (UDP-galactose:ceramide galactosyl-transferase), and macrophage/microglia activation by *Cd68* in spinal cord at the HEP by RT-qPCR. mRNA quantification is given relative to wild type controls *Galc^+/+^ Ripk1^+/+^*, matched for age and sex, with *Gapdh* (glyceraldehyde-3-phosphate dehydrogenase) as the internal control. There was no difference in the expression of these genes between *Galc^twi-2J/twi-2J^ Ripk1^K45A/K45A^* and *Galc^twi-2J/twi-2J^ Ripk1^K45A/+^* (*P* > 0.05); myelin/oligodendrocytes were similarly lost and macrophage/microglia equally activated (Fig. 7D). We then examined proteins Cnpase (2′, 3′-cyclic nucleotide 3′-phosphodiesterase) and β-tubulin-III in sciatic nerve for myelin and axon abundance by immunoblotting, and found both proteins profoundly reduced in *Galc^twi-2J/twi-2J^ Ripk1^K45A/K45A^* and *Galc^twi-2J/twi-2J^ Ripk1^K45A/+^*mice (Fig. 7E).

Possible alterations in the amounts of transcript *Tnf* and related molecules involved in downstream signalling, inflammation and cell death, were also sought in the spinal cord by RT-qPCR. The levels of *Tnf*, *Tnfrsf1*, *Ripk1*, *Rpk3*, *Mlkl*, *Casp8* and *Casp3* were not significantly different between *Galc^twi-2J/twi-2J^ Ripk1^K45A/K45A^* and *Galc^twi-2J/twi-2J^ Ripk1^K45A/+^*mice (*P* > 0.05; Fig. 7F). Disease-associated microglia genes *Cst7*, *Ch25h*, *Apoe* and *Trem2* were equally expressed in these animals, as were *CatD*, *B* and *S* and cytokines *Mip-1α* and *Rantes* (*P* > 0.05). Upregulation of *cIAP2*, but not *cIAP1*, was again replicated in these mice (Fig. 7F). We then examined changes in Ripk1, cathepsin D and caspase-8 proteins in the brain stem of the same animals; they were increased but did not differ between genotypes *Galc^twi-2J/twi-2J^ Ripk1^K45A/K45A^* and *Galc^twi-2J/twi-2J^ Ripk1^K45A/+^* (Fig. 7G).

To explore autophasome/lysosome function, we examined expression of *Atg5*, *LC3* and *Sqstm1* in spinal cord by RT-qPCR: while there was an upward trend for *Sqstm1* in tissue from the *Galc^twi-2J/twi-2J^ Ripk1^K45A/K45A^* and *Galc^twi-2J/twi-2J^ Ripk1^K45A/+^* mice, the changes were not significantly different from healthy controls (*P* > 0.05; Fig. 7H) and were in line with our earlier results from *Galc^twi-2J/twi-2J^*. LC3B and p62 proteins were assessed by immunoblotting in brain stem for direct comparison with results from those of our inbred twi-2J strain, and densitometry quantifications of the LC3B-I and LC3B-II species taken. The LC3B-II/LC3B-I ratio was increased 2-fold in *Galc^twi-2J/twi-2J^ Ripk1^K45A/K45A^* and *Galc^twi-2J/twi-2J^ Ripk1^K45A/+^* samples compared with healthy controls (Fig. 7I) and thus resembling the changes observed in *Galc^twi-2J/twi-2J^* mice. Expression of p62 and ubiquitin1 and their distribution in sciatic nerve, spinal cord and brain sections was investigated by IHC, but no differences in the expression of these molecules was seen compared with mutant *Galc^twi-2J/twi-2J^ Ripk1 ^+/+^* mice (data not shown).

Early in this work we sought to establish whether Ripk1 is phosphorylated at residue serine 166 in twitcher and SD tissues: no convincing signals were detected by immunoblotting with an antibody raised against p-Ripk1 (S166). The creation of our *Galc^twi-2J/twi-2J^ Ripk1^K45A/K45A^* mouse line provided an opportunity to revisit this analysis, as we would not expect Ripk1 S166 to be phosphorylated in these animals, serving as a negative control. We extracted the protein soluble fraction with RIPA buffer from sciatic nerve and spinal cord from Twi-2J and from SD spinal cord, and subsequently dissolved the insoluble fraction by addition of 6M urea. Both fractions were immunoblotted with a p-RIPK1 (S166) antibody (Cell Signaling Technology # 31122) used extensively by others. A species of about the right size was found in all genotypes, *Galc^twi-2J/twi-2J^ Ripk1^K45A/K45A^*, *Galc^twi-2J/twi-2J^*, *Galc^+/+^* and SD mice (Supplementary Material, Fig. S2a–c). Most studies where the p-RIPK1 (S166) antibody has been applied with convincing results use cultured cells in experiments where almost uniform cellular responses would be expected. Given that our findings show Ripk1 expression in a relatively few activated macrophage/microglia, immunoblotting may lack the sensitivity needed to detect activated Ripk1. To increase the probability of detecting phosphorylation at S166, we first immuneprecipitated total Ripk1, and then immunoblotted with p-Ripk1 (S166) antibody (# 31122): this stratagem was also unavailing, but after washing the membrane and re-probing with a different Ripk1 antibody that had been successfully used to detect total Ripk1, a species of the correct size was observed (Supplementary Material, Fig. S2d). To determine whether Ripk1 was ubiquitinated, re-probing with an ubiquitin1 antibody revealed species of sizes greater than 74 kDa, but there was not a clear-cut difference between the genotypes (Supplementary Material, Fig. S2d). Overall these results suggest that Ripk1 kinase is unlikely to be activated in either the twitcher or the Sandhoff mouse.

From these studies in the twitcher strain, we conclude that Ripk1 kinase function has no primary role in driving or compensating for the effects of GALC deficiency in Krabbe disease.

## Discussion

Krabbe disease, a classical inborn error of sphingolipid metabolism, remains an enigma since we have little understanding of its pathogenesis. We have explored the relationship between GALC deficiency and neuroinflammation in biochemically and genetically coherent models of the disorder in mice: as in affected humans, twitcher mice overproduce the metabolite psychosine which exacerbates the acute injury in the nervous system (17). However, although psychosine is a known cytotoxin, the molecular pathways that orchestrate the death of myelin-producing cells and induce the neuroinflammatory features of this disease are not understood.

The pathological hallmarks of Krabbe disease include infiltration of the nervous system by multinucleated globoid cells. These resemble the pathognomonic Gaucher cells and both originate from haematopoietic, mononuclear-macrophage precursors (50,51). Interlinked biochemical and cytological phenomena occur: impaired lysosomal recycling of the parent glycospingolipid in Gaucher disease activates acid ceramidase with the release of β-glucosylsphingosine—an epimeric glycoform of psychosine in Krabbe disease (52). Since both metabolites partition in aqueous as well as lipid phases, psychosines are distributed widely and can be found in neural and visceral tissues as well as in plasma. Exposure of cells of macrophage lineage to quasi-pathological concentrations of psychosines, arrests cytokinesis and induces phenocopies of multinucleate globoid or Gaucher cells (53). Thus, psychosines contribute to the overt cytological as well as other pathological features of these particular and biochemically related sphingolipidoses (54,55).

Ripk1/Ripk3 associated with marked neuroinflammation in animal models of diseases such as multiple sclerosis (56), amyotrophic lateral sclerosis (57) and Alzheimer’s disease (34) and their inhibition improved the disease phenotype. Therefore, since increased expression of Ripk1 and Ripk3 was detected in the brains of twitcher and neuronopathic Gaucher mice, and the course of experimental neuronopathic Gaucher disease was modified in mice with ablation of Ripk3 (44), we investigated the involvement of Ripk1 and related molecules in the pathogenesis of Krabbe disease.

### Ripk1 expression correlates with Krabbe disease severity and progression

Given that Vitner *et al.* reported increased immunoreactive Ripk1 and Ripk3 protein signals in brain homogenates from mutant twitcher mice, we investigated whether expression of these molecules was spatiotemporally regulated. At the HEP, Ripk1 protein was unevenly distributed in the neuraxis in twitcher (twi-2J): greater abundance in spinal cord and brain stem compared with cerebrum and cerebellum, immediately suggesting an association with disease severity. Onset of demyelination in this model occurs 10–20 days after initial myelination, and according to studies in twitcher mice by Taniike and Suzuki, the spinal cord, brain stem and cerebellar white matter demyelinate first—before P20 (58). We found that Ripk1 overexpression correlates with central nervous system (CNS) structures naturally rich in myelin and which are among the first to demyelinate in twitcher.

We studied two twitcher mouse strains that model human Krabbe disease: the classic twi-2J mutation, commonly known as twitcher, is caused by a nonsense mutation at codon 339 in *Galc*, with no detectable Galc in tissues. The twi-5J harbours a missense mutation, E130K, that occurs in human infantile Krabbe disease; the Galc precursor polypeptide is present but catalytically inactive. The twi-5J mutation appears to cause a more severe phenotype: whereas only 25% of homozygous twi-5J are alive at P24, almost all twi-2J survive to P35. The reason for the difference in severity is unclear, but Potter *et al.* could not find a correlation between the amounts of psychosine and demyelination in twi-5J, raising doubts as to the contribution of this metabolite in the pathophysiology and the validity of the ‘psychosine hypothesis’ (32). The distinctive phenotypes of the strains is striking, and we speculated as to whether Ripk1 expression might correlate with the increased severity in the twi-5J strain. Comparison of sciatic nerves at P24 revealed no gross differences between the strains. Contrary to expectation, quantification of Ripk1 in spinal cord at P24 showed a higher content in twi-2J tissue. However, the ratio between mutant and wild type for each respective strain was similar. This suggests that Ripk1 expression is determined by unknown genetic factors that differ between the strains. In this connection, the genetic backgrounds for twi-2J and twi-5J are C57BL/6J and BXD32/TyJ, respectively. When we compared twi-5J at P24 and our longest survivors at P35 with age-matched twi-2J, the cerebrum of twi-5J had a greater burden of Ripk1 at both ages; the amount of Ripk1 at P24 in twi-5J was higher than at P35 in twi-2J. Ripk1 was not elevated above healthy controls in twi-2J cerebrum at P24. The increased abundance of Ripk1 in twi-5J compared with twi-2J in this brain structure suggests an association with disease pathology: its expression parallels increases in cathepsin D, caspase-8 and gliosis. We thus conclude that Ripk1 abundance correlates with disease severity and progression in mouse models of Krabbe disease.

### Molecules intimately connected with Ripk1 signalling are also upregulated in the sphingolipidoses Krabbe and SD

The complex functions of the multifaceted Ripk1 molecule are gradually being unravelled (37,59–62). We examined the spinal cord of twitcher as a paradigm site of disease which undergoes degeneration: at the transcriptional level, and starting at the earliest age studied, P11, *Tnf* was gradually upregulated, indicating an early response to pathological signal/s occurring before the onset of demyelination. Expression of the cognate receptor, *Tnfr1*, mirrored this abnormality. *Ripk1* transcript abundance together with other core elements of the necroptosome, *Ripk3* and *Mlkl,* also increased as the disease progressed. After ligation by FasL, the death receptor Fas activates caspase-8, mediated by the adaptor molecule Fadd. *Fas* and *Fadd* transcripts were upregulated, as was *Casp8,* which increased over time, but the effector of apoptosis, *Casp3*, was only significantly elevated at the HEP (P40). *cFlip_L_*, whose transcription is regulated by transcription factor NF-κB, and restrains the apoptotic response upon caspase-8 activation, was elevated but only at the HEP. Taken together our findings show that not only is Ripk1 abnormally expressed in the twitcher mouse but all elements of the ripoptosome/necroptosome studied are overrepresented. However, it is not possible to determine whether activation of these components leads inexorably to disease or these processes are being halted. To examine the relative roles these components have in the pathogenesis of Krabbe disease, it would be necessary to disrupt their functions and examine the individual effects as well as those that might occur in combination.

Unlike Krabbe disease, the lysosomal disorder SD, principally affects neurones. The finding by Vitner and colleagues (44), that Ripk1 abnormal expression appeared to be specific to Gaucher and Krabbe diseases was of great interest, as these two disorders share pathological features that are distinct from other sphingolipidoses. Furthermore, abolition of Ripk3 in a model of neuronopathic Gaucher disease improved the condition. On the basis of these observations, a unique role for Ripk1/Ripk3 underpinning some of the molecular mechanisms of disease appeared to be justifiable and we predicted that a similar stratagem would improve the phenotype in twitcher. The Sandhoff mouse was used here to serve as a negative control but contrary to expectations, Ripk1 was also elevated in the brain of this animal—changes accompanied by increases in cathepsin D and caspase-8 expression. The findings prompted reconsideration of the transcriptional expression of core elements of the ripoptosome/necroptosome in Sandhoff mice: at the HEP, all elements studied were significantly upregulated, but to a lesser extent than in twitcher. It remains unclear as to the reason our results differ from those reported (44). In this respect, it should be pointed out that Cougnoux and colleagues also demonstrated upregulation of some of these molecules in the lysosomal disease Niemann-Pick C1 (63). We conclude that upregulation of elements of the ripoptosome/necroptosome system is not unique to Gaucher and Krabbe disease, but it is likely to be a common feature of lysosomal diseases—or at least those, the majority, in which neurodegenerative manifestations occur.

### Ripk1 expression accompanies DAM and neuroinflammatory features in Krabbe and SD

A recently discovered microglia subtype named DAM associates with neurodegenerative disease, and its distinctive transcriptome and function emerges from the microglia pool as the condition deteriorates (21). Investigations in a model of Alzheimer disease suggested that Ripk1 regulates DAM gene expression, including *Cst7* and *Ch25h* (34)—findings that prompted our exploration of Ripk1 as a potential modulator of these genes and DAM-associated markers in Krabbe and Sandoff disease. Not only was *Cst7* expression highly induced at the transcriptional and translational level in these disorders but in twitcher, *Cst7* transcription increased with advancing disease and concomitant upregulation of the three examined cathepsins. DAM markers Apoe and Trem2 were similarly increased, and Gpnmb, which has also been identified in the brains of mouse models of Gaucher disease as well as in patients after developing Alzheimer’s disease (22), was increased several 1000-fold in the spinal cord of twitcher.

It has been postulated that the primary function of DAM is to mitigate disease outcome; by enhancing the phagocytic activity of microglia and restraining cytokine production and secretion, thereby suppressing damaging inflammatory responses (22). However, this appears contrary to findings by Ofengeim *et al.* in Alzheimer’s disease, modelled in the APP/PS1 mouse. They provided evidence of Ripk1 catalytic activity in microglia acting as a regulator of *Cst7* and *Ch25h* expression: induction of *Cst7* mediated by Ripk1 impaired lysosomal function and thus undermined the phagocytic capacity of microglia resulting in increased disease severity. Inhibition of Ripk1 kinase function, by interbreeding APP/PS1 with the kinase-dead Ripk1 D138N mouse, reduced *Cst7* and *Ch25h* expression in microglia, and ameliorated amyloid burden, expression of inflammatory cytokines Tnfa and Il1β, with improved neurological function (34).

*cIAP1* and *cIAP2* are differentially expressed in twitcher, *cIAP2* but not *cIAP1* is elevated from the earliest time point studied, P11, and therefore before the start of demyelination. The expression of *cIAP2* increased over time, correlating with disease severity and progression. This suggests that the phenotype of activated macrophage/microglia in Krabbe disease is pro-inflammatory, characteristic of the polarised M1 phenotype, as opposed to the anti-inflammatory tissue-repair M2 phenotype (46).

Ripk1 signalling downstream of inflammatory molecules such as Tnf is well-acknowledged. Stimulation of cells with Tnf induces the expression of cytokines such as *Mip-1α*, which were increased in all murine models of disease we investigated here. Activation of astrocytes as well as microglia occurs in most neurodegenerative disorders, and as shown by Liddelow *et al.* the processes of these two cell types are linked. Indeed, secretion of cytokines Il-1α, Tnf and C1q by microglia is necessary and sufficient to induce the activation of astrocytes; once activated, astrocytes can kill neurones and oligodendrocytes (48). We found that these cytokines are upregulated in twitcher and SD mice, which together with the DAM gene expression signature, Ripk1 and cIAP2 overabundance is evidence of a heighten state of inflammation. Taken together, our data overwhelmingly reflects a prominent state of neuroinflammation, which is particularly pronounced in Krabbe disease.

### Proteosomal/autophagosomal/lysosomal flux defects in the sphingolipidoses Krabbe and SD

The assertion by Ofengeim *et al.* that inhibition of the lysosome/UPS system in microglia causes a rapid induction of Ripk1 kinase activation and the suggestion that Ripk1 might regulate lysosomal function in these cells is of general importance—and highly relevant to diseases in which the lysosome compartment is principally affected. Ofengeim *et al.* showed accumulation of p62 and LC3II in a microglia cell line transfected with Cst7, but the evidence that Ripk1 kinase function mediates this phenomenon is indirect. Although an increase in total Ripk1 protein content in Cst7-transfected cells was detected by immunoblotting, no specific staining for p-Ripk1 (S166) was demonstrated, nor was the presence of P62/LC3II accumulation established in microphages staining positive for Ripk1 and Cst7 by IHC in the APP/PS1 mouse model (34). Clearly more research is needed to clarify the nature of the relationship between Ripk1 and the lysosome.

We investigated abnormalities in the UPS/autophagosomal/lysosomal systems in our disease models, and identified pathological accumulation of p62 and LC3II by immunoblotting and IHC staining. Of note, we found p62 in cells which were Oligo2- or NeuN-positive in twitcher but only co-stained with NeuN cells in SD mice. Many p62 labelled cells were also SMI32-positive in twitcher, a sign of neuronal/axonal degeneration, and reminiscent of the axonopathy described by Castelvetri *et al.* (64). Our findings are generally supportive of research studies (65,66), and with the impaired lysosomal flux described in SD by Boland *et al.* (67). Aggregates of p62 and LC3II have been identified in a mouse deficient in the sphingolipid activator saposine A, which cooperates with Galc to hydrolyse key galactolipids, and in a model of combined deficiencies of saposin A and saposin B. p62 or LC3II deposits were not found in animals deficient in saposin B alone; saposine B being required for lysosomal breakdown of the sulphated galactosphingolipid, sulphatide (68). Diseases in which the normal function of the lysosome is compromised are accompanied by inflammation, and in those with neurodegenerative manifestations neuroinflammation is a cardinal feature. However, the underlying molecular mechanisms that link a dysfunctional lysosomal compartment to neuroinflammation remain to be defined. One plausible explanation is that signals from damaged/stressed cells, caused by a disabled lysosome, are released and detected by microglia, which upon stimulation initiate an inflammatory response to dampened down the injury. Unlike in transient disorders, the damage cannot be contained long term in these diseases, which indeed increases over time. Thus, the inflammatory cycle is perpetuated and amplified and likely to cause further injury to neighbouring tissue.

The UPS and autophagy are separate pathways for proteolytic degradation but are highly regulated and at times interdependent; under conditions of high cellular stress they co-operate to ensure efficient turnover of misfolded proteins and salvage of critical nutrients. The multifunctional protein p62 is involved in the proteasomal degradation of ubiquitinated proteins but serves as a receptor for cell cargo destined for disposal by autophagy, of which it is also a substrate. That most cells positive for p62 co-stain with ubuquitin1 in twitcher and the SD mouse, indicates selective autophagy. In this type of autophagy, molecules are marked for degradation with specific labels to ease recognition by autophagy receptors and to facilitate their co-aggregate into large structures for phagophore formation. Selective autophagy is important for substrates that cannot be degraded by proteasomes (69). Our results add further support to the observation that abnormal UPS/autophagosome/lysosome traffic and degradation are features of Krabbe and SD. Unexpectedly, we found no clear evidence of similar abnormalities in phagocytic cells, microglia and astrocytes, whose function it is to scavenge degraded myelin, axons and dead cells. Important questions remain about the performance of these enzymatically defective cells when processing complex debris in the diseased nervous system that contains partially digested macromolecular substrates that are refractory to digestion. Recently, Weinstock *et al.* addressed this very question in macrophages that migrate to nerves as a response to demyelination in the absence of Galc in a Schwann cell-specific manner. They demonstrated the need of macrophages for autonomous expression of Galc to facilitate myelin degradation, but in the absence of Galc, as is the case in Krabbe disease, the presence of undegraded myelin worsens the peripheral neuropathy. However, when Galc-competent macrophages are present, the phenotype is attenuated (19). Related findings have been described for ASA (arylsulfatase A), the enzyme defective in the sister disease metachromatic leukodystrophy (70).

### Ripk1 localizes to macrophage/microglia with an activated/phagocytic phenotype and ablation of its kinase function does not alter the disease course in twitcher

In twitcher mice as in other models, Ripk1 was upregulated in macrophage/microglia but small amounts might be also present in other cell types. The Ripk1-positive macrophage/microglia had an activated phenotype and formed clusters, reminiscent of those around amyloid plaques in the APP/PS1 mouse; in twitcher, we presume the clusters are formed around degenerating myelin and cell debris. We found no clear evidence that expression of Ripk1 in these cells causes dysfunction to the autophagosomal/lysosomal/UPS system, as co-localization of Ripk1 and ubiquitin1/p62 was only identified in a small number of cells.

To investigate the contribution of Ripk1 to Krabbe disease pathogenesis, we crossed the twitcher with a Ripk1 kinase-dead (K45A) mouse and bred the offspring to homozygosity. As described, the natural course of disease was unaffected and it was clear that the kinase activity of Ripk1 neither impacts development of Krabbe disease in this model, nor its relentless progression. Analysis of DAM gene expression, including abundance of *Cst7* and *Ch25h*, cytokines and molecules cognate to Ripk1 signalling remained unchanged.

The different outcomes for twitcher and the APP/PS1 mouse when Ripk1 catalytic activity is abolished is striking. One possible explanation is that in Krabbe disease the autophagosmal/lysosomal system is inherently defective due to deficiency of Galc, and in this context the negative contribution of Ripk1 kinase function might not be discernible. Another possibility is that the kinase function of Ripk1 is not activated in Krabbe disease, and no evidence of Ripk1 kinase activation was found in twitcher mice. This contrasts with reported pathological activation of Ripk1 in the APP/PS1 mouse, in which inhibition of the activity would indeed be expected to improve outcome.

We have identified abnormal Ripk1 expression in activated macrophage/microglia in twitcher which explains Ripk1 association with progression of disease severity, but the function executed by Ripk1 in these cells and its effect on the nervous system remains unknown. The genetic tools and knowledge now available enable the pathological role played by Ripk1 to be further explored as it has in other diseases. Given the complex and tight cellular regulation of Ripk1 expression, we contend that the marked Ripk1 upregulation in activated macrophage/microglia here noted in experimental Krabbe disease is likely to reflect as yet ill-understood but pathologically important biological processes.

## Materials and Methods

### Animals

The natural mutant twitchers, twi-2J [#000978, C57BL/6J-*Galc^twi-2J^* (71)] and twi-5J [#003613, BXD32/TyJ-*Galc^twi-5J^*/J (72)], and the Sandhoff (SD) knockout [#002914, B6;129S4-*Hexb^tm1Rlp^*/J (73)] were obtained from The Jackson Laboratory (Bar Harbor, ME, USA). The *Ripk1^K45A^* knock-in mouse was a kind gift from GlaxoSmithKline (43). All strains are maintained by heterozygous matings, except for the Galc/Ripk1double mutant, which is maintained as heterozygotes for *Galc^twi-2J^* and homozygotes *for Ripk1^K45A^*. Studies were conducted using protocols approved under license by the U.K. Home Office (Animals Scientific Procedures Act, 1986). Galc twi-2J, twi-5J, Hexb, Ripk1(K45A) genotyping was determined by PCR essentially as described elsewhere (43,45,74,75). Mice had access to food and water *ad libitum* and were provided with nutritional supplements (Transgel; Charles River Laboratories) on the cage surface. The approved HEP applied to mice throughout this study was defined as the loss of between 10 and 15% from the maximum achieved weight. Animals were killed at any time if they developed clinical signs such as visceral enlargement, tumours and self-inflicted injuries.

### Tissue processing

Mice were killed by CO2 asphyxiation and organs snap-frozen in optimum cutting temperature medium on dry-ice, or given a lethal dose of pentobarbital and transcardially perfused with ice-cold phosphate buffered saline (PBS), followed by cold PBS containing 4% paraformaldehyde (pH 7.4). Perfused tissue was post-fixed in the same fixative for a few hours, followed by either incubation in 30% sucrose overnight at 4°C for cryoprotection, then frozen and stored at −80°C, or processed for wax embedding instead. Fifteen micrometre coronal sections were cut from frozen blocks and mounted on Superfrost glass slides and stored at −80°C. Three to 5 μm sections were cut from wax-embedded tissue, mounted on glass slides and stored at RT.

### Histological staining

For IHC staining of non-perfused tissue with fluorescent antibodies sections were warmed at RT for 30 min, washed in PBS three times at RT for 5 min and fixed in cold PBS containing 4% paraformaldehyde (pH 7.4) for 10 min before staining. Bright-field immunohistochemistry was performed as previously described (75). Wax-embedded sections were deparaffinised; incubated first in xylene, followed by washes in different percentages of ethanol in water and finishing with a tap water rinse. We carried out antigen retrieval by incubating slides at 95–100°C for 20 min in 10 mm trisodium citrate, pH 6.0. Slides were left to cool at RT for at least 30 min. Sections were blocked/permeabilised in 20% new equine serum (NES), 0.2% Triton X-100/PBS for 1 h at RT. Primary antibodies were diluted in 2% NES, 0.2% Triton X-100/PBS and incubated at 4°C overnight. Slides were washed in PBS three times for 5 min with slight shaking, and incubated with fluorescent secondary antibodies diluted in 2% NES, 0.2% Triton X-100/PBS for 1–2 h at RT, washed in PBS and mounted with Prolong Gold anti-fade mounting medium (Invitrogen). Confocal microscopy was performed using a Leica Sp5 ultra-high speed inverted confocal microscope and images analyzed with Fiji software. Primary antibodies: mouse anti-Gfap (1/100, Sigma #G3893), rabbit anti-Iba1 (1/500,Wako #01919741), rabbit anti-LC3 (1/200, Novus Biologicals #NB100–2331), rat anti-Mac2 (1/250; Cedarlane #CL8942AP), mouse anti-Mbp (1/1000, Calbiochem #NE1018), mouse anti-NeuN (1/100, Millipore #MAB377), mouse anti-non-phosphorylated neurofilament heavy chain (1/1000, Covance #SMI32), rabbit anti-Olig2 (1/500, Millipore #AB9610), rabbit anti-p62 (1/1000, MBL #PM045B) and mouse anti-p62 (1/1000, abcam #ab56416) and mouse anti-Ubiquitin1 (1/250, Millipore #MAB1510). Secondary antibodies Alexa-488 and -568 (Molecular Probes) from different species were used at 1/200 dilution, as well as donkey anti-mouseAlexa-680 (1/500, Invitrogen/Thermo Fisher #A32788).

For combined mRNA ISH and IHC staining, animals were given a lethal dose of pentobarbital and transcardially perfused. Dissected tissue was treated as described previously, and 15 μm sections cut with a cryostat, mounted on Superfrost glass slides and stored at −80°C. The ISH RNAscope Multiplex FL v2 procedure was performed following the manufacture’s recommendations for fixed-frozen sections. We first tested the methodology on our tissue sections with negative and positive control probes, # 320871 (to bacterial *DapB*) and # 320881 (a 3-plex probe mix to mouse *Polr2a*-C1, *Ppib*-C2 and *Ubc*-C3), respectively. Our target mRNA probe Mm-Ripk1-C1 (Advanced Cell Diagnostics/Bio-Techne # 464511) was used undiluted and the signal was developed with dye Opal™ 570 (Akoya Biosciences, #FP1488001KT) diluted 1/1500. After completion of the ISH protocol, sections were washed in PBS and blocked in 5% NES, 0.2% Triton X-100/PBS for 1 h at RT, then washed again in PBS and incubated with different primary antibodies in 2% NES, 0.2% Triton X-100/PBS overnight at RT. Slides were washed in PBS and incubated with fluorescent secondary antibodies diluted in 2% NES, 0.2% Triton X-100/PBS for 1–2 h at RT, washed in PBS and mounted with Prolong Gold anti-fade mounting medium. Slides were stored in the dark at 4°C and left to dry for several days before visualization by confocal microscopy. Twenty-five to 29 *z*-stack confocal images were taken and analyzed with Fiji software.

### Western blotting

Tissue protein extraction was performed in ice-cold lysis buffer: RIPA (radioimmunoprecipitation assay) buffer, either from Fisher Scientific (Pierce #89900) or Millipore (#20–188), containing protease inhibitors (cOmplete Mini, EDTA-free; Roche Diagnostics #11836170001) and phosphatase inhibitors (Sigma #P0044). For extraction of insoluble protein fraction, a second extraction was performed with lysis buffer containing 6M urea. We used the Pierce Thermo scientific kit #23227 for measuring protein concentration, following recommended instructions.

For polyacrylamide-gel electrophoresis and immunoblotting, after reduction and denaturation in sodium dodecyl sulphate and 4% β-mercaptoethanol, 5–200 μg of protein extracts from tissue or from cells 293T transfected with a plasmid expressing mouse caspase-8 (OriGene Technologies #MC200404) and Neuro2a chloroquine-treated (Novus Biologicals #NBP2-49688) were heated at 90°C and run in 4–15% linear gradient gels (161–1122; Bio-Rad) or in 8, 10, 12 and 15% gels made in-house, proteins were transferred onto 0.45 μm PVDF membranes (Millipore #IPV00010). Western blots were processed with primary antibodies: mouse anti-β-actin (1/5000; Sigma #A5316), rabbit anti-Caspase-8 (1/1000, Cell Signaling Technology #4790), goat anti-Cathepsin D (1/500, Santa Cruz Biotechnologies #sc-6486), mouse anti-Cnpase (1/2000, Sigma #C5922), rabbit anti-Cst7 (1/1000, a kind gift from Prof Colin Watts, University of Dundee UK, #2080), rabbit anti-Gapdh (1/10 000, Sigma #G9545), rabbit anti-LC3B (1/2000, Novus Biologicals #NB100-2220), mouse anti-Mbp (1/1000; Calbiochem), rabbit anti-p62 (1/1000, MBL#PM045), mouse anti-Ripk1 (1/1000, BD Transduction laboratories #610458) and rabbit anti-Ripk1 (1/1000, Cell Signaling Technology #3493), Mouse anti-α-tubulin (1/20 000, Sigma #T6074), mouse anti-β-tubulin III (1/1000, Sigma #T2200). Horseradish peroxidase conjugated secondary antibodies were: goat anti-mouse (1/5000, DakoCytomation #P0447), rabbit anti-goat (1/5000, DakoCytomation #P0449) and goat anti-rabbit (1/5000, DakoCytomation #P0448). Blots were developed with Amersham ECL™ western blotting Analysis System (GE Healthcare #RPN2109) following manufactures’ recommendations. Blots were often stripped and re-probed with other antibodies. We used the stripping method recommended by Abcam. In brief, membranes were incubated in stripping buffer containing glycine, SDS and Tween 20 pH 2.2 twice for 10 min with some shaking and then washed several times in TBS-T (0.1% Tween 20 in Tris-buffered saline).

### Immunoprecipitation with antibody to Ripk1

Two hundred microgram of protein was mixed with 2 μg of mouse anti-Ripk1 antibody [BD Transduction Laboratories, clone: 38/RIP (RUO), #610458] and incubated with rotation at 4°C overnight. Then, 50 μl of Protein A/G Ultralink Resin (Thermo Scientific #53133) was added and incubated with rotation at 4°C for 2 h. Complexes were collected by centrifugation at 2500 g for 3 min at 4°C, and unbound lysate removed. Complexes were washed five times with 1 ml 1×TBS (10×TBS, Bio-Rad #170-6435) and collected by centrifugation at 2500 g for 3 min at 4°C. Immunocomplexes were eluted with 50 μl of 2XNuPAGE LDS sample buffer (Invitrogen #NP0007) and incubated at 95°C for 5 min. Supernatant was collected after centrifugation at 2500 g for 3 min and run on an 8% SDS-PAGE gel. Following wet-transfer of proteins onto membrane and after blocking with Pierce protein-free T20 blocking buffer (Thermo Scientific #37571) for 1 h at RT, the membrane was first incubated with rabbit anti-p-RIPK1 (S166; 1/500, Cell Signaling Technology #31122) at 4°C overnight, followed by our standard immunoblotting protocol. Once the membrane was developed, it was stripped with Restore western blot stripping buffer (ThermoFisher Scientific #21059) and re-probed with rabbit anti-Ripk1 (1/1000, Cell Signaling Technology #3493). A third stripping and re-probing was carried out with mouse anti-Ubiquitin1 (1/1000, Millipore #MAB1510). Secondary antibodies were goat anti-rabbit (1/5000, DakoCytomation #P0448) and goat anti-mouse (1/5000, DakoCytomation #P0447).

### Real-time PCR

Total RNA was extracted from 30 mg of tissue using the RNeasy kit (Qiagen #74104) and first-strand cDNA was synthesized by reverse transcription of 200–500 ng of RNA in a 20-μl total volume with random primers following the manufactures’ recommendations (Applied Biosystems, kit #4368814). Relative quantitation was performed by real-time PCR on an ABI fast 7500 PCR System (Applied Biosystems). One microliter of different dilutions of reverse transcription reaction was mixed with 300 nmol of each primer and Power SYBR green PCR master mix (Applied Biosystems #4367659) to a final volume of 20 μl. Thermal cycling conditions were: one cycle at 95°C for 10 min and 40 cycles at 95°C for 15 s followed by 60°C for 1 min. Primers were: Apoe (Apolipoprotein E) forward (F): 5′-CTCCCAAGTCACACAAGAAC-3′ and reverse (R): 5′-TTGCGTAGATCCTCCATGTC-3′; Atg5 (Autophagy related 5) (F): 5′-ACACACTTGGAGATCTCCTC-3′ and (R): 5′-TATCTGGGTAGCTCAGATGC-3′; C1qa (Complement component 1, q subcomponent) (F): 5′-TGCTGACCATGACCCTAGTA-3′ and (R): 5′-AAAACCTCGGATACCAGTCC-3′; C3 (Complement component 3) (F): 5′-TGCACCAAGTACTTGGGAGA-3′ and (R): 5′-GTGTTCTTGTTGGAGAAGGC-3′; C4b (Complement component 4B) (F): 5′-CTGGAGAAGCTGACCTCTCT-3′ and (R): 5′-GTAGTCATACAGGACAGCAC-3′; Casp3 (Caspase 3) (F): 5′-ACTGTGGCATTGAGACAGAC-3′ and (R): 5′-AGCTTCAGCATGCTGCAAAG-3′; Casp8 (Caspase 8) (F): 5′-AGTGAGCGAGTTGGAATTGAG-3′ and (R): 5′-CATGGTCCTCTTCTCCATTTC-3′; CatB (Cathepsin B) (F): 5-AAATCAGGAGTATACAAGCATGA-3′ and (R): 5′-GCCCAGGGATGCGGATGG-3′; CatD (Cathepsin D) (F): 5′-CTGAGTGGCTTCATGGGAAT-3′ and (R): 5′-CCTGACAGTGGAGAAGGAGC-3′; CatS (Cathepsin S) (F): 5′-ACCTACCAAGTGGGCATGAACGAT and (R): 5′-TCGGGAAATTCTCAGAGCACCCAT-3′; Cd68 (cluster of differentiation 68) primer set: Mm-Cd68–1-SG QuantiTect primer assay (QT00254051; Qiagen); cFlip (cellular FLICE-like inhibitory protein) (F): 5′-CTGTGCACAGCAGACGTATC-3′ and (R): 5′-CACCACTGTTCCACGCATAC-3′; Cgt (UDPgalactose:ceramide galactosyltransferase) (F): 5′-AGTTTCCA AGACCAACGCTGC-3′ and (R): 5′-TGTTCCTGAGCACCA CTTACC-3′; Ch25h (Cholesterol 25-hydroxylase) (F): 5′-TCCACTCACAGACTTGTGCC-3′ and (R): 5′-TGCCCAGCATTTTGTCCCAG-3′; cIAP1 (cellular Inhibitor of Apoptosis Protein 1) (F): 5′-CAGAAGACGTTTCAGGCTTG-3′ and (R): 5′-GCACAGTCCCCTTGATTGTC-3′; cIAP2 (cellular Inhibitor of Apoptosis Protein 2) (F): 5′-GGACATTAGGAGTCTTCCCA-3′ and (R): 5′-AGCGCAGTCTTTGCACACGA-3′; Cst7 (Cystatin F) (F): 5′-TGACTTCCAAACCAACCCTG-3′ and (R): 5′-GTATCACAGCTGCAGTCTTG-3′; Fadd (Fas-associated protein with death domain) (F): 5′-AGATCTGCAGGTGGCATTTG-3′ and (R): 5′-TTCTCAGCATTCTTCCAGAC-3′; Fas (Fas receptor) (F): 5′-CAGGATGACCCTGAATCTAG-3′ and (R): 5′-CTGTCATGCATGATCTCATC-3′; Gapdh (Glyceraldehyde-3-phosphate dehydrogenase) (F): 5′-TGTGTCCGTCGTGGATCTGA-3′ and (R): 5′-TTGCTGTTGAAGTCGCAGGAG-3′; Gfap (glial fibrillary acidic protein) (F): 5′-AGTAACATGCAAGAGACAGAG-3′ and (R): 5′-TAGTCGTTAGCTTCGTGCTTG-3′; Gpnmb (Glycoprotein nonmetastatic melanoma protein B) (F): 5′-GCACCTACTGTGTGAATTTC-3′and (R): 5′-GCAAGATGGTAACCATGGTG-3′; Il1α (Interleukin 1 alpha) (F): 5′-TGATGAAGCTCGTCAGGCAG-3′ and (R): 5′-CGACGAGTAGGCATACATGT-3′; LC3B (Microtubule-associated protein 1 light chain 3 beta) (F): 5′-TGGAAGATGTCCGGCTCATC-3′ and (R): 5′-TGATGAGCTCGCTCATGTTC-3′; Mbp (Myelin basic protein) (F): 5′-TTCCAGGAGTCATTGCTGCTA-3′ and (R): 5′-TGGAGTTCTGCACCATTGAT-3′; Mip-1a (Macrophage inflammatory protein 1 alpha) forward (F): 5′-TCTGTACCATGACACTCTGC-3′ and (R): 5′ AATTGGCGTGGAATCTTCCG 3′; Mlkl (Mixed lineage kinase domain-like) (F): 5′- TCACAGATCTCCAGTTACCATC-3′ and (R): 5′-ACGCAAGATGTTGGGAGAATCG −3′; Rantes (regulated on activation normal T cell expressed and secreted) (F): 5′-AGT GCT CCA ATC TTG CAG TC-3′ and (R): 5′-AGCTCATCT CCAAATAGTTG-3′; Ripk1 (Receptor-interacting serine–threonine kinase 1) (F): 5′-AGTCGAGACTGAAGGACACAGCACT-3′ and (R): 5′-TCCAGCAGGTCACTGGATGCCAT-3′; Ripk3 (Receptor-interacting serine–threonine kinase 3) (F): 5′-CTTGAACCCTCCGCTCCTGC-3′ and (R): 5′-AATCTGCTAGCTT GGCGTGG-3′; Sqstm1 (Sequestosome 1) (F): 5′-AGCTGCTCTTCGGAAGTCAG-3′ and (R): 5′-ATGTGTCCAGTCATCGTCTC-3′; Tnf (Tumor necrosis factor) (F): 5′-CCACCACGCTCTTCTGTCTA-3′ and (R): 5′-CACTTGGTGGTTTGCTACGA-3′; Tnfrsf1 (Tumor necrosis factor receptor superfamily, member 1a) (F): 5′-TAGAGAGCTCAGCCAGCGCT-3′ and (R): 5′-GTGGCTTCCGTGGGAAGAAT-3′; Trem2 (Triggering receptor expressed on myeloid cells 2) (F): 5′-CCACCTCCATTCTTCTCCTC-3′ and (R): 5′-GGTCCAGTGAGGATCTGAAG-3′.

We performed RT-qPCR on three animals per group and in triplicate, with Gapdh as the internal control. The analysis was calculated with the Delta–delta Ct method, and graphically represented as the mean ± SD (standard deviation). Significance was analyzed with the Student’s *t*-test against wild type mice, matched for age and sex and a *P*-value ≤ 0.05 (^*^) considered statistically significant.

### Statistics

Kaplan–Meier survival curves were analyzed with the log-rank equivalent to the Mantel–Cox test. The statistics were analyzed with one-way ANOVA and Bonferroni multiple post hoc comparisons using GraphPad Prism v5.0 (GraphPad Software). Values with *P* ≤ 0.05 were considered significant. The Student’s *t*-test was applied when comparing two samples. ^*^  *P* ≤ 0.05; ^*^^*^  *P* ≤ 0.01; ^*^^*^^*^  *P* ≤ 0.001 and ^*^^*^^*^^*^  *P* ≤ 0.0001. ImageJ was used for densitometry measurements.

## Supplementary Material

Supplementary_Material_Fig_S1_tif_ddab159

Supplementary_Material_Fig_S2_tif_ddab159
