# Supplementary figures and images for: Expression of Ripk1 and DAM genes correlates with severity and progression of Krabbe disease
Source: Hum Mol Genet. 2021 Jun 25;30(22):2082–99. doi: 10.1093/hmg/ddab159 (PMC8561423; doi:10.1093/hmg/ddab159)

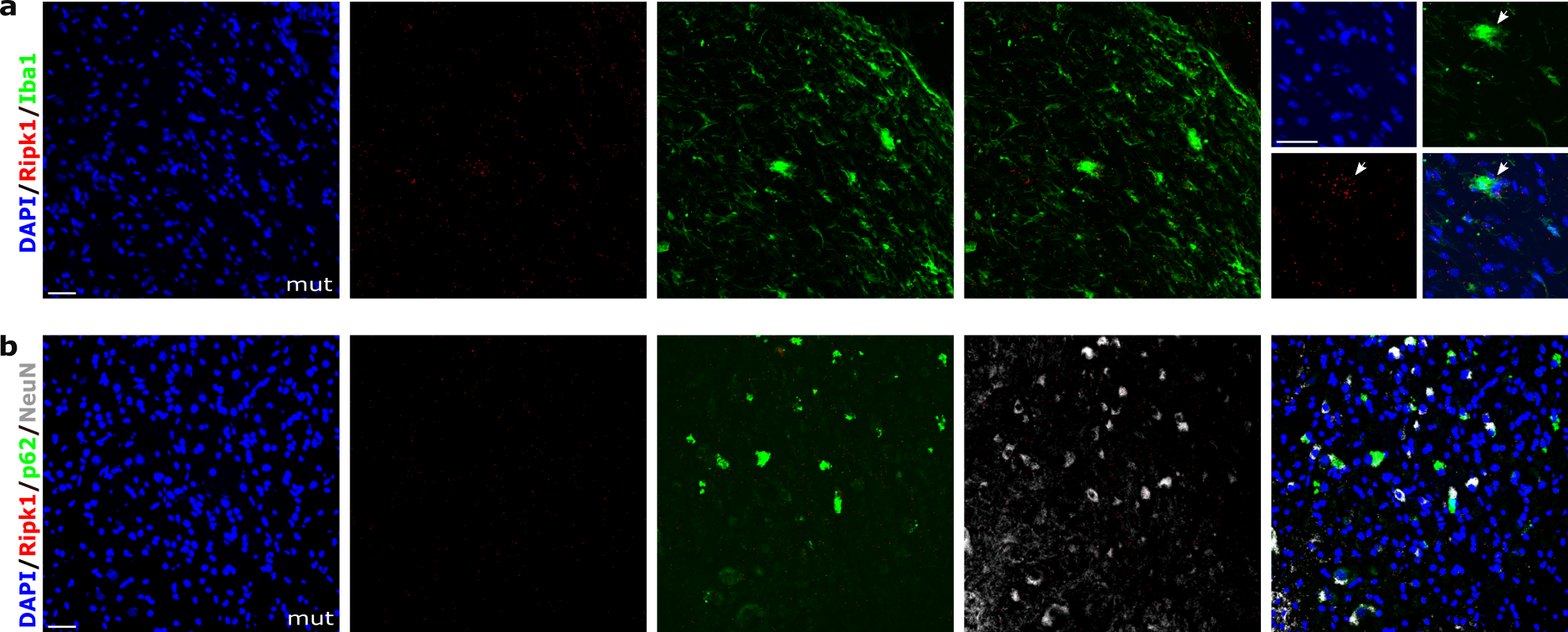

Supplement: Supplementary_Material_Fig_S1_tif_ddab159 [file supplementary_material_fig_s1_tif_ddab159.zip › Supplementary_Material_Fig_S1_tif_ddab159.tif]

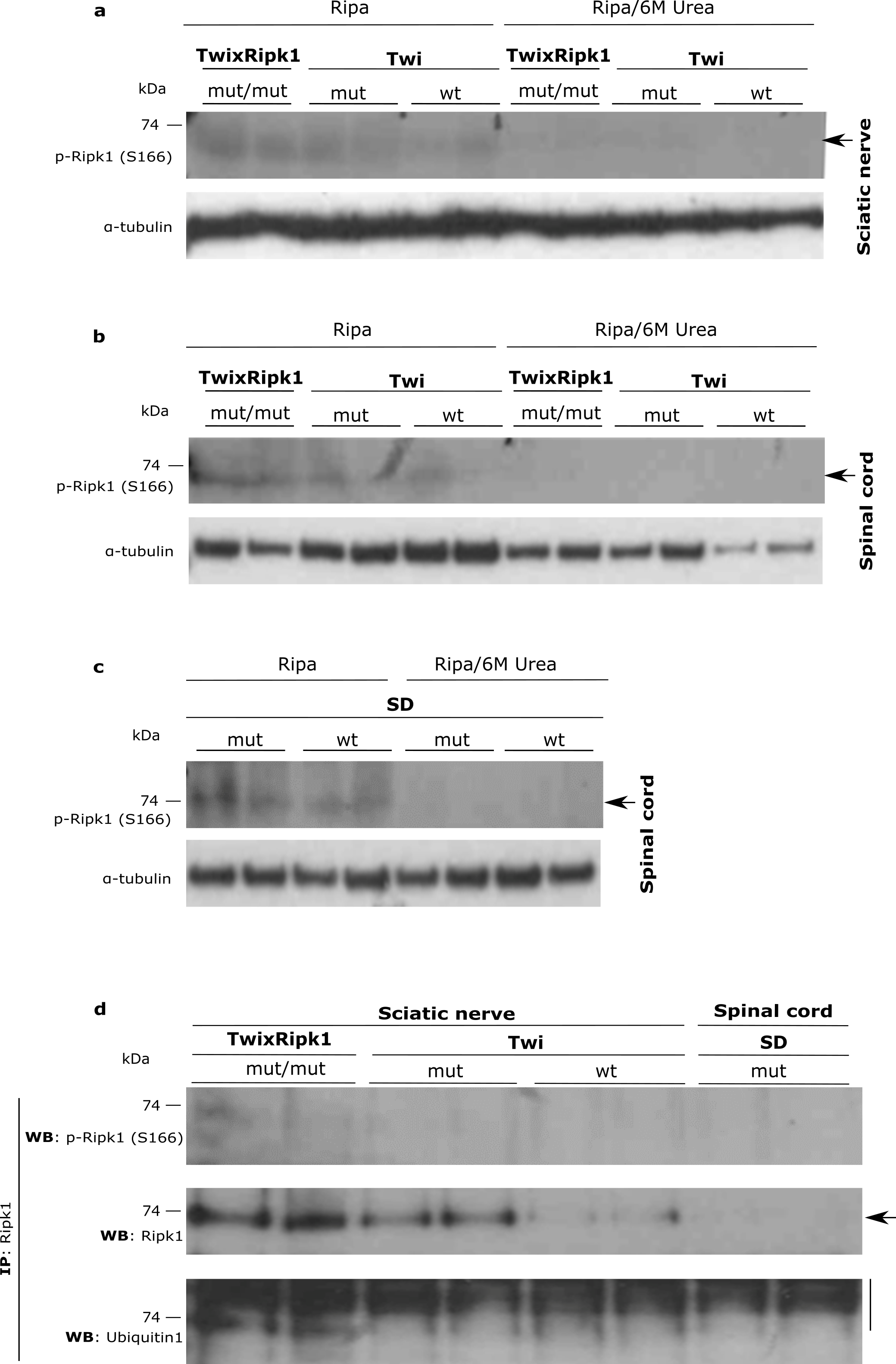

Supplement: Supplementary_Material_Fig_S2_tif_ddab159 [file supplementary_material_fig_s2_tif_ddab159.zip › Supplementary_Material_Fig_S2_tif_ddab159.tif]
